# The effects of a physical and cognitive training intervention vs. physical training alone on older adults’ physical activity: A randomized controlled trial with extended follow-up during COVID-19

**DOI:** 10.1371/journal.pone.0258559

**Published:** 2021-10-13

**Authors:** Tiina Savikangas, Timo Törmäkangas, Anna Tirkkonen, Markku Alen, Roger A. Fielding, Miia Kivipelto, Timo Rantalainen, Anna Stigsdotter Neely, Sarianna Sipilä

**Affiliations:** 1 Gerontology Research Center and Faculty of Sport and Health Sciences, University of Jyväskylä, Jyväskylä, Finland; 2 Department of Medical Rehabilitation, Oulu University Hospital, Oulu, Finland; 3 Nutrition, Exercise Physiology, and Sarcopenia Laboratory, Jean Mayer USDA Human Nutrition Research Center on Aging, Tufts University, Boston, Massachusetts, United States of America; 4 Population Health Unit, Finnish Institute for Health and Welfare, Helsinki, Finland; 5 Division of Clinical Geriatrics, Center for Alzheimer Research, NVS, Karolinska Institutet, Stockholm, Sweden; 6 Institute of Clinical Medicine/Neurology, University of Eastern Finland, Kuopio, Finland; 7 Neuroepidemiology and Ageing Research Unit, School of Public Health, Imperial College London, London, United Kingdom; 8 Department of Social and Psychological Studies, Karlstad University, Karlstad, Sweden; 9 Department of Psychology, Umeå University, Umeå, Sweden; Prince Sattam Bin Abdulaziz University, College of Applied Medical Sciences, SAUDI ARABIA

## Abstract

**Background:**

Executive functions underlie self-regulation and are thus important for physical activity and adaptation to new situations. The aim was to investigate, if yearlong physical and cognitive training (PTCT) had greater effects on physical activity among older adults than physical training (PT) alone, and if executive functions predicted physical activity at baseline, after six (6m) and twelve months (12m) of the interventions, one-year post-intervention follow-up and an extended follow-up during COVID-19 lockdown.

**Methods:**

Data from a single-blinded, parallel-group randomized controlled trial (PASSWORD-study, ISRCTN52388040) were utilized. Participants were 70–85 years old community-dwelling men and women from Jyväskylä, Finland. PT (n = 159) included supervised resistance, walking and balance training, home-exercises and self-administered moderate activity. PTCT (n = 155) included PT and cognitive training targeting executive functions on a computer program. Physical activity was assessed with a one-item, seven-scale question. Executive functions were assessed with color-word Stroop, Trail Making Test (TMT) B-A and Letter Fluency. Changes in physical activity were modeled with multinomial logistic models and the impact of executive functions on physical activity with latent change score models.

**Results:**

No significant group-by-time interaction was observed for physical activity (p>0.1). The subjects were likely to select an activity category higher than baseline throughout the study (pooled data: B = 0.720–1.614, p<0.001–0.046). Higher baseline Stroop predicted higher physical activity through all subsequent time-points (pooled data: B = 0.011–0.013, p = 0.015–0.030). Higher baseline TMT B–A predicted higher physical activity at 6m (pooled data: B = 0.007, p = 0.006) and during COVID-19 (B = 0.005, p = 0.030). In the PT group, higher baseline Letter Fluency predicted higher physical activity at 12m (B = -0.028, p = 0.030) and follow-up (B = -0.042, p = 0.002).

**Conclusions:**

Cognitive training did not have additive effects over physical training alone on physical activity, but multicomponent training and higher executive function at baseline may support adaptation to and maintenance of a physically active lifestyle among older adults.

## Introduction

Physical activity is crucial for older adults’ health, functioning and well-being [1, 2]. Despite the well-known and numerous benefits, physical activity declines with increasing age [3] and a large proportion of older adults are physically inactive [4, 5]. Physical inactivity has been considered a severe challenge worldwide and defined as a pandemic for almost ten years ago [6]. Group exercise interventions may be an effective tool to increase older adults’ physical activity [1, 7, 8], since they enable e.g., social support, perceived health benefits, feeling better and getting up, out and going [9]. The positive effects of training programs on physical activity tend, however, to be short-lived [7]. Thus, more research is needed on what intervention strategies lead to sustained changes in physical activity.

Among older adults, better executive functions–higher order cognitive processes required for planned and goal-oriented behavior [10]–have been recognized as potential predictors of higher physical activity, exercise adherence and maintenance [11–14]. Current research suggests that fundamental facets of executive functioning, including working memory, behavioral inhibition and task switching, underlie self-regulation [15]. Executive function may also influence the capability to choose a behavior that may require acute exertion and discomfort but bring benefits in the long term, instead of a behavior that brings acute pleasure but is associated with negative long-term consequences [16].

A large body of research suggests that both physical [17, 18] and cognitive training interventions can improve executive function [19]. Furthermore, a recent meta-analysis suggests that combining physical and cognitive training may lead to greater increases in executive functioning than physical training alone [20], and the evidence is complemented by our previous study [21]. In a 12-month randomized controlled trial, we found that some aspects of executive functions improved more in older adults, who participated in targeted executive functions training in addition to physical training compared to those, who were assigned to physical training alone [21]. Even though the transfer effects of executive functions training on everyday life behavior are not clear [22], complementing exercise interventions with executive functions training may improve executive functions and thus facilitate better adherence to a physically active lifestyle. It has, however, not been studied, if targeted executive functions training in addition to physical training can support adherence to a structured physical training intervention or maintenance of physical activity during the post-intervention follow-up period.

Executive functions also facilitate the adaptation to novel and challenging situations [23], and may therefore be of special importance for maintaining physical activity in situations, where habitual physical activity and exercise routines are challenged. Currently, the world is facing the Coronavirus Disease 2019 (COVID-19) pandemic, and concerns have arisen that it may lead to worsening of the physical inactivity pandemic [24]. During the outbreak of the COVID-19 in the spring 2020, all public sports facilities were closed, group activities quitted, and gatherings of more than ten people were prohibited in Finland. Furthermore, people over 70 years were obligated to stay in self-quarantine and to avoid physical contacts with others. In these exceptional circumstances, a person with higher executive functions may find new ways to be physically active. In a recent study, executive functioning deficits were associated with negative changes in physical activity during COVID-19 among younger adults [25], but research is lacking among older adults.

This is an exploratory post-hoc analysis of the PASSWORD-study, a 12-month randomized controlled trial with a one-year post-intervention follow-up [21, 26]. In the study, some aspects of executive functions improved more among older adults participating in physical and cognitive training intervention compared to those attending physical training alone, but gait improved similarly in both study groups [21]. The present study includes also an extended follow-up during COVID-19 lockdown, which was declared in mid-March 2020, when the original 12-month follow-up period of the PASSWORD-study was about to end.

The aim of this study was to investigate, whether 12-months physical and cognitive training intervention had greater effects than physical training alone on physical activity among older adults, who did not meet physical activity recommendations prior to the intervention. We hypothesized that physical activity improved more and was maintained better after the interventions, when continuous supervision and support from the study personnel were ended, in the combined training group. Additionally, we investigated if executive functioning was associated with physical activity during the interventions, follow-up, and the COVID-19 pandemic. We hypothesized that higher executive function predicted higher physical activity.

## Methods

### Study design

This is an exploratory post-hoc analysis of the PASSWORD-study (“Promoting safe walking among older people”, ISRCTN52388040), a two-arm, parallel-group, single-blinded randomized controlled trial conducted at the Gerontology Research Centre at the Faculty of Sport and Health Sciences, University of Jyväskylä, Finland [21, 26]. The study had a one-year post-intervention follow-up, and the present analysis also includes an extended follow-up during COVID-19 lockdown. During the post-intervention measurements, participants were encouraged to continue a physically active lifestyle and received information about senior gyms and training groups in the city of Jyväskylä. Other support or supervision was not provided by the study personnel after the intervention.

The main outcome of the PASSWORD was 10 meters maximal walking speed. Study design and recruitment process have been described in detail [26] and main results have been published [21]. Sample size calculations were performed a-priori for the main outcome of the PASSWORD-study, i.e., 10 meters maximal gait speed, as reported by Sipilä et al [21, 26]. A priori power analysis was not conducted for this exploratory study.

The reporting of this trial followed the Consolidated Standards of Reporting Trial guidelines (CONSORT; S1 File). This study has been carried out in accordance with Declaration of Helsinki and the study protocol was approved by the Ethics committee of the Central Finland Health Care District (14/12/2016, ref: 11/2016; 24/4/2020, ref: 11U/2016). All participants signed a written informed consent before baseline measurements. The trial protocol, including analysis plans for the primary outcome measure, was prospectively registered on the ISRCTN registry (52388040). The original research plan for the PASSWORD-study is provided as S2 File. Major changes considering the present study were exclusion of three months measurements due to lack of resources and addition of the COVID-19 questionnaire. These changes were approved by the ethics committee.

### Participants

**Fig 1:**
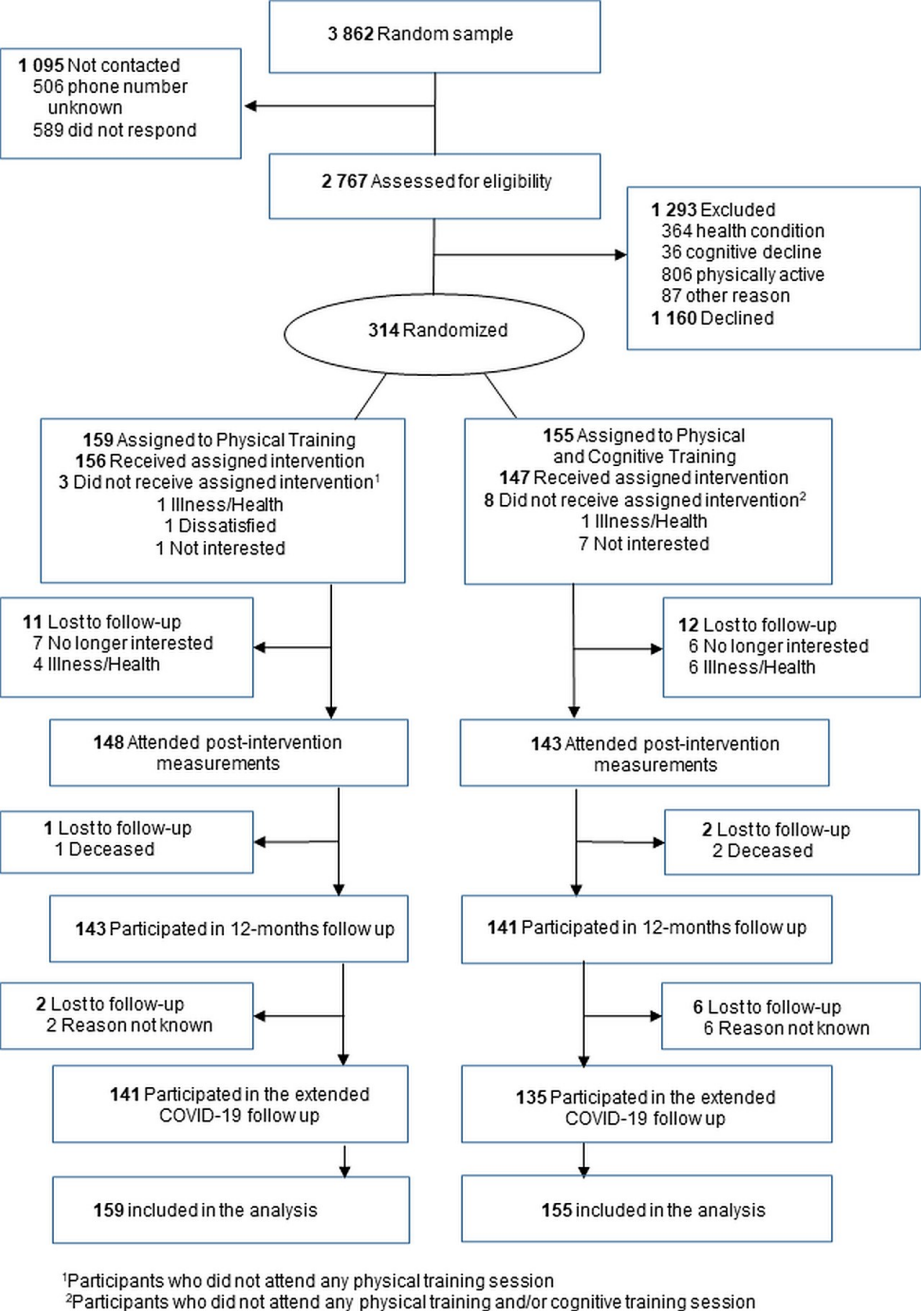
Flow chart of the study.

Community-dwelling, 70–85 years old men and women, who lived in the city of Jyväskylä, Finland, were recruited between January 2017 and March 2018. Participants were eligible for the study, if they did not meet the physical activity recommendations of the time (less than 150 min of moderate activity/week and no regular resistance training), were able to walk 500 m without assistance, and scored ≥ 24 points in the Mini Mental State Examination (MMSE) test. Exclusion criteria were: severe chronic condition and/or medication or behavioral factor that could have compromised participation in the study, difficulties in communication due to severe vision or hearing problem, excessive alcohol consumption, and other family member participating in the PASSWORD-study. An initial random sample of 3862 people was drawn from the Finnish National Population Registry. After a screening interview over phone and clinical screening of the inclusion and exclusion criteria at the laboratory, 314 participants were recruited to the study (Fig 1).

### Randomization and blinding

Participants were randomized in a 1:1 ratio to receive either physical and cognitive training (PTCT, n = 155) or physical training alone (PT, n = 159). A computer-generated random allocation sequence, created by a senior biostatistician, was used to allocate subjects into training groups in randomly varying blocks of two and four, stratified by sex and age group (70–74, 75–79, 80–85). Randomization and assigning participants to intervention was done by a senior researcher, who did not participate in the data collection or the interventions. Investigators collecting the data and supervisors of physical training groups were blinded to the group allocation, and participants were asked not to mention their group to the investigators or supervisors.

### Interventions

Interventions have been described in detail in previous publications [21, 26]. Briefly, both study groups participated in a multicomponent physical training program. The intervention was adapted from the physical activity recommendations for older adults of the time [27], our earlier study [28], and the LIFE-study [29, 30]. Physical training was divided into several training periods, which varied in terms of training specificity, volume and intensity. Training loads and difficulty were increased progressively. Two supervised 45–60 minutes training sessions per week were organized: one concentrating on walking and dynamic balance and the other on resistance and balance training. Supervised walking sessions consisted of a warm-up, including walking at self-selected speed and progressive dynamic balance exercises, and continuous walking for 10–20 minutes at a target intensity of 13–15 on the Borg scale [31]. The resistance exercise sessions started with a 10-minute warm-up and balance exercises. Thereafter, 8–9 resistance exercises targeting lower body, trunk and upper body muscles were performed with machines utilizing air pressure technology (http://www.hur.fi/en). Participants received also a progressive home exercise program with target training frequency of 2–3 times per week, including strengthening exercises for the lower limbs, balance training and stretching. In addition, participants were instructed to accumulate 150 minutes per week of moderate intensity aerobic activity in bouts of at least 10 minutes. Physical training sessions were supervised by trained research assistants, who were Master’s degree students of sport and health sciences or physiotherapist students. The average duration of the intervention was 51 weeks and on average 46 supervised resistance training sessions were provided. Walking exercises were started after adoption phase to physical training and had a summer break, resulting in an average of 36 supervised walking sessions provided.

The cognitive training (CT) was performed on an in-house developed web-based computer program (iPASS), which was modified from a program previously used in other studies [32, 33]. CT was started at a university computer class, and supervised by trained research assistants with, at least, psychology as a minor subject. CT targeted executive functions, i.e., inhibition, shifting and updating of working memory. Four different tasks were practiced during each training session. The tasks were organized into two blocks: Block 1 included letter updating, predictable set-shifting, spatial working memory maintenance, and color inference tasks to train inhibition, whereas Block 2 included spatial updating, unpredictable set-shifting, spatial working memory maintenance, and number inference tasks to train inhibition. Target training frequency was 3–4 times a week. Participants were allowed to start CT at home after 2–3 group sessions, if they had a computer and necessary computer skills. Participants were also given the possibility to train at the University computer class and/or specific locations provided by the City of Jyväskylä. Support for computer skills was available during given training times at the university and other specific locations. The first weeks of the intervention consisted of an adoption phase to physical training, and the average length of the cognitive intervention was 46 weeks.

Adherence and adverse events have been reported previously, and no between-group differences were observed [21]. As reported earlier by Sipilä et al [21], approximately 40% of the participants reported some adverse events, and 10% reported intervention-related adverse events or symptoms. These were mostly transient non-severe pain and/or discomfort in the joints and/or muscles of the lower body.

### Outcome measures

#### Physical activity

Physical activity was assessed with a questionnaire at baseline (BL), after six (6m) and twelve months (12m) of the interventions, one-year post-intervention follow-up (FU) and during the COVID-19lockdown. The time from FU to COVID-19 varied between two weeks and sixteen months (median six months) depending on the recruitment date of the participant. A single-item, seven-option response scale question about the current physical activity participation was utilized (“Which of the following descriptions best corresponds to your physical activity at the moment?”). The response options were: (0) I do not move more than is necessary in my daily chores, (1) I go for casual walks and engage in light outdoor recreation 1–2 times a week, (2) I go for casual walks and engage in light outdoor recreation several times a week, (3) I engage 1–2 times a week in brisk physical activity (e.g. yard work, walking, cycling) to the point of perspiring and some degree of breathlessness, (4) I engage several times a week (3–5) in brisk physical activity (e.g. yard work, walking, cycling) to the point of perspiring and some degree of breathlessness, (5) I do keep-fit exercises several times a week in a way that causes rather strong shortness of breath and sweating during the activity, and (6) I participate in competitive sports and maintain my fitness through regular training [34]. Participants were asked to select the highest response option that corresponded to their current physical activity. Due to no responses in category 6 and very few responses in category 5, categories 4 and 5 were combined for the analyses.

**Fig 2:**
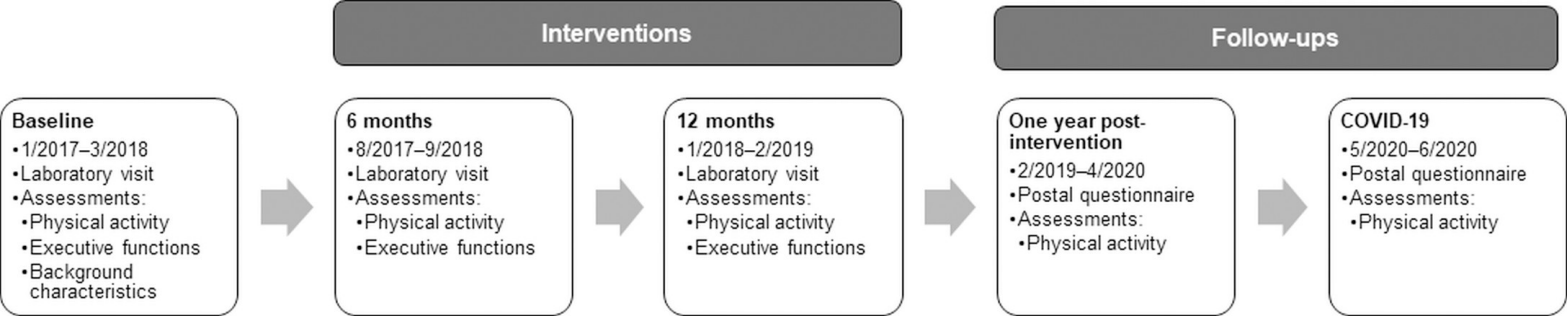
Timeline of the study.

At BL, 6m and 12m participants returned the questionnaire at the research center during the laboratory assessments. FU questionnaire was posted to each participant with a prepaid envelope one year after his/her post-intervention measurement and returned by mail. Reminder calls were made, if necessary. If a participant returned the questionnaire with missing data, it was completed interviewer-assisted over telephone. Data collection was completed in April 2020. COVID-19 questionnaire was sent with a prepaid return envelope in the end of April 2020. A reminder text message was sent approximately a month later and a reminder and a new questionnaire with a new prepaid envelope were posted in beginning of June, if necessary. At that time point, the state of emergency was still in force and people aged over 70 years were recommended to self-quarantine, even though the institutional services were reopening. Data collection was completed in the end of June 2020. Timeline of the study is shown in Fig 2.

The questionnaire used in this study has acceptable test-retest reliability but limited validity among middle-aged women [35]. This kind of a single-item questionnaire can be sensitive enough to detect statistically significant group-by-time interactions in change of physical activity due to a physical training program [36].

#### Executive functions

Executive functions were assessed at baseline, and after six and twelve months of the interventions with Color-Word Stroop Test (Stroop) [37], Trail Making Test B–A (TMT B–A) [38] and Letter Verbal Fluency Test [39]. Stroop was used to assess response inhibition [40]. First, participants were asked to read aloud 72 color words printed in black ink (control). Second, they were asked to read aloud the color of 72 printed X’s (congruent). Third, participants were shown a list with 72 color words printed in incongruent color (e.g., the word BLUE printed with red ink) and asked to read aloud the ink color while ignoring the word itself (incongruent). The time to complete each test condition was recorded, and the time difference between the congruent and incongruent conditions was calculated. The smaller the difference, the better the performance.

TMT B–A was utilized to assess cognitive flexibility and set shifting [40]. In TMT A participants were asked to draw a line connecting numbers 1–25 in sequential order, and in TMT B to draw a line connecting alternately numbers 1–13 and letters A–L in ascending order, i.e., from 1 to A, A to 2, 2 to B etc. TMT B–A was calculated as the time difference between completing TMT A and TMT B, smaller difference indicating better performance.

Letter fluency was utilized to assess updating [41]. Participants were asked to verbally generate as many unique words beginning with the letters P, A and S as possible in three separate one-minute trials. A sum score of the three trials was calculated. Higher score indicates better performance.

#### Background characteristics

Sex and age at baseline were drawn from national population registry. Weight (kg) and height (m) were measured by the study nurse, and body mass index (BMI, kg/m^2^) was calculated. Fat percent was assessed with dual-energy X-ray absorptiometry (DXA, LUNAR Prodigy, GE Healthcare).

Highest education (college/university degree vs. no college/university degree), marital status (married/cohabiting vs. unmarried/widowed/in a relationship, but not living together), smoking status (never, i.e. smoked less than 100 cigarettes during lifetime, vs. former vs. current), self-perceived current health (very good/good vs. average/poor), perceived difficulties in outdoor mobility (five-scale range from no difficulties to not capable to move outdoors even with assistance, re-categorized as no difficulties vs. at least minor difficulties) and prolonged musculoskeletal pain in any part of the body hindering physical activity (no vs. yes) were drawn from a comprehensive questionnaire. Physical function was assessed with Short Physical Performance Battery (SPPB, total score range 0–12, higher score indicates better performance), including five-time chair rise, habitual walking speed over four meters and standing balance tests [42].

### Statistical analysis

Descriptive statistics are shown as means and standard deviations (SD) for continuous variables and frequencies (no.) and percentages (%) for categorical analyses. Differences between participants who dropped out and those who did not were assessed with Pearson’s Chi squared test or Fisher’s exact test for categorical variables and independent samples t-test for continuous variables.

**Fig 3:**
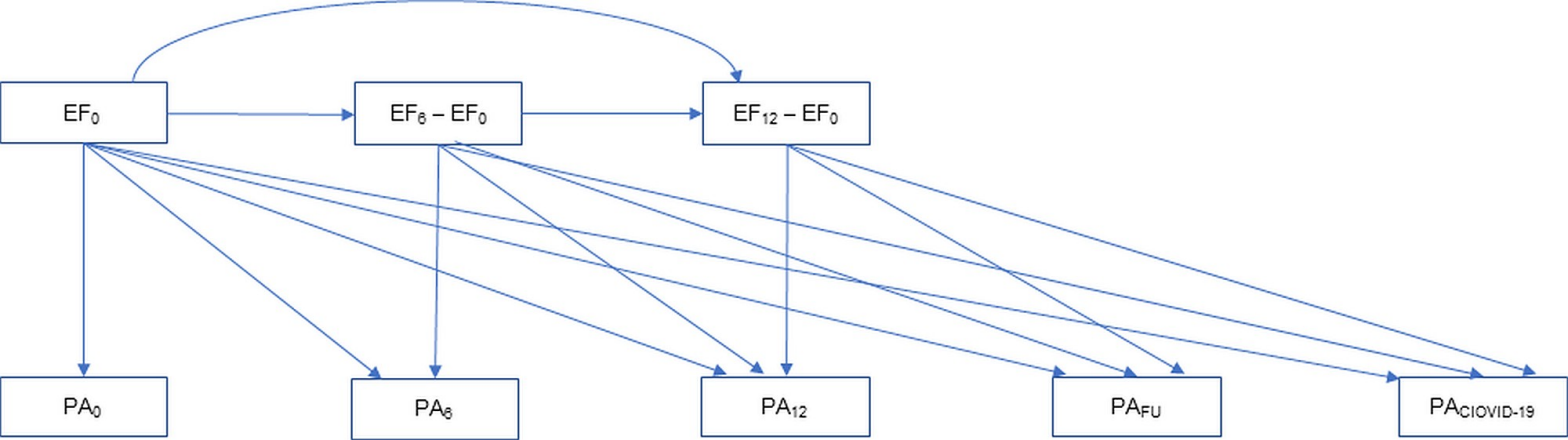
Change score model. Joint and separate change score models were created for the three executive function (EF) variables to assess the impact of changes in executive functions on concurrent and subsequent physical activity (PA) measurement (BL = baseline, 6m = six months, 12m = twelve months, FU = one year post-intervention follow-up).

Initially, two multinomial logistic longitudinal path models (MLLPM) were used to model changes in the physical activity outcome: 1) intervention-control two-group model including time (BL, 6m, 12m, FU and COVID-19), and 2) one-group (pooled) model including only time factor. Wald tests were used for comparison of changes in physical activity across intervention groups (main effects of group, time and group × time interaction) following the intention-to-treat principle. Next, we augmented to models with additional change score models for each of the three executive function variables to assess the impact of changes in executive functions on concurrent and subsequent physical activity measurement (Fig 3). These analyzes were conducted separately and joint for the three executive function variables.

In addition to those who dropped out, information on physical activity was missing from one participant at 6m, two participants at 12m and one participant during FU. One participant did not complete the TMT B–A test at BL and 12m. Based on the assumption that incomplete data was generated through the missing-at-random mechanism, we used the maximum likelihood estimator adapted for incomplete data (for details, see Muthén & Muthén, 1998–2004, Appendix 6) [43] in all models.

The time between FU and COVID-19 measurements was calculated from self-reported response dates on the questionnaires. Missing dates were imputed by hand from questionnaire mailing dates. Participants were expected to have answered the questionnaires within two weeks from posting the questionnaires.

Descriptive statistics were computed and attrition analyses performed with IBM SPSS Statistics 26 (SPSS Inc., Armonk, NY). Multinomial logistic models, and joint MLLPM and latent change score models were constructed using Mplus, version 7.4. Statistical significance level was set at 0.05 for all analyses.

## Results

### Participant characteristics

Participant characteristics (n = 314, mean age 74.5 ± 3.8 years, 60% women) are presented in Table 1. Nearly half of the participants perceived their current health as good or very good at the baseline, but 40% reported prolonged pain hindering physical activity during the past six months, and one of five participants reported at least minor difficulties in outdoor mobility. Mean SPPB score was 10.

**Table 1:** Participant characteristics by physical and cognitive training (PTCT) and physical training (PT) groups.

	PTCT (n = 155)	PT (n = 159)
Age, mean (SD), y	74.4 (3.9)	74.5 (3.8)
Women, no. (%)	96 (62)	92 (58)
Body mass index, mean (SD), kg/m^2^	28.0 (4.9)	27.9 (4.5)
Fat percent, mean (SD); n_PTCT_ = 154	36.4 (8.3)	35.9 (8.1)
Marital status, no. (%)		
Cohabiting	102 (66)	97 (61)
Other	53 (34)	62 (39)
Education, no. (%)		
College /university degree	38 (25)	28 (18)
High school or less	117 (76)	131 (82)
Smoking status, no. (%)		
Never smoker	94 (61)	97 (61)
Former smoker	52 (34)	57 (36)
Current smoker	9 (6)	5 (3)
SPPB, mean (SD) ^a^	10.2 (1.5)	10.1 (1.6)
Perceived difficulties in outdoor mobility, no. (%)		
No difficulties	122 (79)	123 (77)
At least minor difficulties	33 (21)	36 (23)
Self-rated health, no. (%)		
Very good/good	73 (47)	68 (43)
Average/poor	82 (53)	91 (57)
Prolonged pain hindering physical activity, no. (%) ^b^	66 (43)	59 (37)
Stroop difference, s, mean (SD) ^c^		
Baseline	45.1 (20.8)	48.1 (28.5)
6 months	34.3 (19.5) (n = 148)	46.5 (25.5) (n = 151)
12 months	34.2 (17.2) (n = 141)	43.6 (20.4) (n = 148)
TMT B-A, s, mean (SD) ^d^		
Baseline	87.2 (55.0)	88.9 (49.4) (n = 158)
6 months	76.5 (47.6) (n = 148)	86.8 (41.8) (n = 151)
12 months	76.3 (57.6) (n = 141)	84.1 (49.4) (n = 147)
Letter fluency, no. of words, mean (SD)		
Baseline	42.3 (13.1)	40.9 (12.9)
6 months	43.2 (13.1) (n = 148)	40.9 (12.1) (n = 151)
12 months	46.7 (14.2) (n = 141)	44.3 (13.3) (n = 148)

Note.

^a^ Short Physical Performance Battery.

^b^ Self-reported, daily or almost daily pain lasting for at least one month during the past six months in neck/shoulders, arms/hands, lower back, hip, knees, or ankles/feet.

^c^ Stroop incongruent–Stroop congruent.

^d^ Trail Making Test B–Trail Making Test A.

Attrition analyses showed that participants who had dropped out at the one year post-intervention follow-up belonged more often to the least physically active category, perceived more often difficulties in outdoor mobility and performed worse in the SPPB test at the baseline than participants who remained in the study (Table 2). Participants who did not participate in the extended follow-up during COVID-19 belonged more often to the least physically active category, were on average older and had lower score in the SPPB test at the baseline than those who remained in the study during all data collecting phases. No statistically significant differences were observed in dropout rates between study groups (p > 0.68) or in other background variables (p > 0.07).

**Table 2:** Attrition analysis by participants’ baseline characteristics.

	One year post-intervention follow-up			COVID-19		
	Respondents (n = 288)	Non-respondents (n = 26)	P	Respondents (n = 276)	Non-respondents (n = 38)	P
Group, no. (%)			0.685			0.731
PTCT	141 (49)	14 (54)		135 (49)	20 (53)	
PT	147 (51)	12 (46)		141 (51)	18 (47)	
Sex, no. (%)			0.837			1.0
Women	173 (60)	15 (58)		165 (60)	23 (60)	
Men	115 (40)	11 (42)		111 (40)	15 (40)	
Age, yrs, mean (SD)	74.3 (3.8)	75.6 (3.9)	0.114	74.3 (3.8)	75.8 (3.8)	**0.020**
BMI, mean (SD), kg/m^2^	27.8 (4.7)	29.4 (4.9)	0.099	27.8 (4.7)	28.9 (4.5)	0.191
Fat percent, mean (SD)	35.9 (8.3)	38.9 (6.7)	0.072	35.9 (8.2)	38.2 (7.9)	0.110
Marital status, no. (%)			0.834			0.722
Married/cohabiting	183 (64)	16 (62)		176 (64)	23 (60)	
Other	105 (36)	10 (38)		100 (36)	15 (40)	
Education, no. (%)			0.453			1.0
College/university degree	59 (20)	7 (27)		58 (21)	8 (21)	
High school or less	229 (80)	19 (73)		218 (79)	30 (79)	
Smoking status, no. (%)			0.934			0.832
Current	13 (4)	1 (4)		13 (5)	1 (3)	
Former	99 (34)	10 (38)		95 (34)	14 (37)	
Never	176 (61)	15 (58)		168 (61)	23 (60)	
SPPB, mean (SD), score ^a^	10.2 (10.5)	9.4 (1.9)	**0.006**	10.2 (1.4)	9.5 (2.0)	**0.023**
Difficulties in outdoor mobility, no. (%)			**0.046**			0.144
No difficulties	229 (80)	16 (62)		219 (79)	26 (68)	
At least minor difficulties	59 (20)	10 (38)		57 (21)	12 (32)	
Self-rated health, no. (%)			0.839			1.0
Very good/good	130 (45)	11 (42)		124 (45)	17 (45)	
Average/poor	158 (55)	15 (58)		152 (55)	21 (55)	
Prolonged pain hindering physical activity, no. (%)			0.836			0.860
Yes	174 (60)	15 (58)		167 (60)	22 (58)	
No	114 (40)	11 (42)		109 (40)	16 (42)	
Stroop difference, mean (SD), s	46.7 (24.7)	46.7 (28.3)	1.0	46.9 (25.1)	45.2 (24.4)	0.694
TMT B–A, mean (SD), s	87.4 (51.2)	94.7 (62.9)	0.501	87.3 (51.2)	93.5 (54.8)	0.490
Letter fluency, mean (SD), score	42.0 (13.1)	37.3 (11.6)	0.078	42.0 (13.2)	38.7 (10.9)	0.139
Physical activity category, no. (%)			**0.047**			**0.039**
0	35 (12)	8 (31)		33 (12)	10 (26)	
1	79 (27)	4 (15)		75 (27)	8 (21)	
2	67 (23)	5 (19)		66 (24)	6 (16)	
3	72 (25)	4 (15)		70 (25)	6 (16)	
4/5	35 (12)	5 (19)		32 (12)	8 (21)	

Note.

^a^ Short Physical Performance Battery, total score, range 0–12, higher score indicates better performance.

^b^ Self-reported, daily or almost daily pain lasting for at least one month during the past six months in neck/shoulders, arms/hands, lower back, hip, knees, or ankles/feet.

^c^ Stroop incongruent–Stroop congruent.

^d^ Trail Making Test B–Trail Making Test A.

### Changes is physical activity

Changes in distribution of physical activity options selected by the subjects did not differ between the study groups at any measurement time point (Table 3).

**Table 3:** Effect estimates in longitudinal linear multinomial model for changes in physical activity category probabilities over time points.

Effect	Parameterization	Est.	S.E.(Est.)	p
Group	PTCT–PT	-0.08	0.34	0.801
Time	6m –BL	1.61	0.29	**<0.001**
	12m –BL	1.34	0.29	**<0.001**
	FU–BL	0.72	0.30	**0.018**
	COVID-19 –BL	1.50	0.29	**<0.001**
Group×Time	PTCT_6m –BL_−PT_6m –BL_	0.16	0.41	0.690
	PTCT_12m –BL_−PT_12m –BL_	0.11	0.42	0.791
	PTCT_FU–BL_−PT_FU–BL_	0.59	0.43	0.171
	PTCT_COVID-19 –BL_−PT_COVID-19 –BL_	0.28	0.42	0.502

Note. Physical activity reference category: highest category (Brisk activity or keep fit exercise several times per week). PT = physical training (Ref), PTCP = physical and cognitive training; BL = baseline (Ref); 6m = six months; 12m = twelve months; FU = one-year post-intervention follow-up. Est = regression coefficient estimate, S.E.(Est) = standard error of regression coefficient estimate. Wald test of time: 42.20 (df = 4), p < 0.0001; Wald test of time-group interaction: 2.66 (df = 4), p = 0.6160.

**Fig 4:**
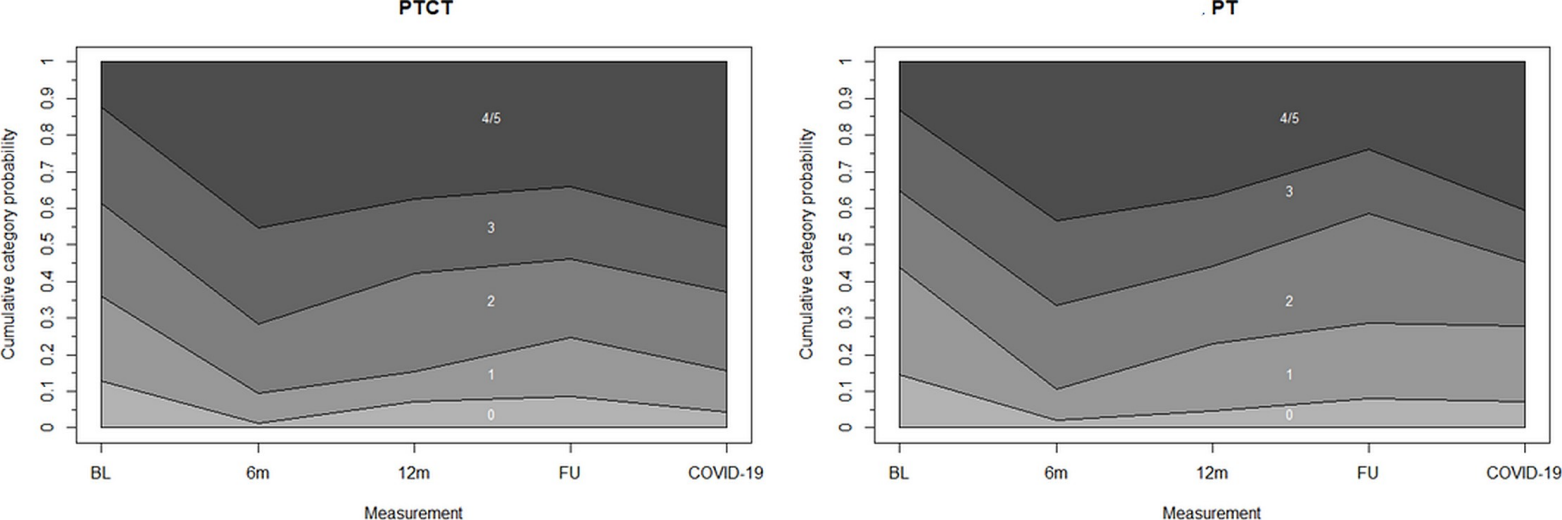
Physical activity category selection probability by study group from the multinomial logistic longitudinal path model. Physical activity categories: 0 = No more than necessary; 1 = Casual walks/light outdoor recreation 1–2 times a week; 2 = Casual walks/light outdoor recreation several times a week; 3 = Brisk physical activity 1–2 times a week; 4/5 = Brisk physical activity or keep fit exercise several times a week. BL = baseline; 6m = six months; 12m = twelve months; FU = one year post-intervention follow-up.

At six months, the subjects in both study groups were more likely to select a physical activity category higher than at baseline and the likelihood attenuated only slightly thereafter, but remained on average statistically significantly higher in all time points following baseline (Fig 4 and Table 3). The proportion of participants who selected a physical activity category higher than at baseline was 64% at six months, 53% at twelve months, 46% one year post-intervention and 56% during COVID-19. The proportion of participants belonging to the highest physical activity category, i.e., reporting several times per week brisk activity or keep fit exercise, increased from 13% at baseline to 44% at six months and 37% at twelve months. At the one-year post-intervention follow-up, 29% of participants were in the highest physical activity category, whereas the proportion was 43% during COVID-19.

### Executive functions predicting physical activity

The joint effects of baseline performance and subsequent changes in the Stroop, TMT B–A and Letter Fluency tests at baseline on physical activity did not differ between the study groups (p for group × EF interaction = 0.138), but we observed a trend towards statistical significance in pooled data (p = 0.055) (Table 4, for details see S1 Table). In separate analysis of each test, the baseline and change effects of Stroop and TMT B–A performance on physical activity did not differ by study group (p for group × EF interaction > 0.3 for both). In the pooled data (one-group model) we found significant associations for Stroop (p = 0.003) and TMT B–A (p = 0.040) predicting physical activity. As for the Letter Fluency performance, the likelihood ratio test indicted that statistically significant group × EF interactions were observed (p = 0.026), suggesting the predictive effect was statistically significant only in the training combination-specific two-group model. Therefore, for Stroop and TMT B–A, we examined the time point-specific impact of performance on physical activity category probabilities using pooled data, while for Letter Fluency we examined the training groups separately (two-group model).

**Table 4:** Tests on effect constraints for executive functions on physical activity.

	Two groups						One group		
	EF → PA			EF × group → PA			EF → PA		
	χ^2^	df	p	χ^2^	df	p	χ^2^	df	p
Joint Stroop, TMT, LF	90	72	0.070	45	36	0.138	51	36	0.055
Stroop	38	24	**0.032**	13	12	0.384	30	12	**0.003**
TMT	35	24	0.072	13	12	0.371	22	12	**0.040**
LF	41	24	**0.016**	23	12	**0.026**	20	12	0.064

Note. ‘EF → PA’ tested if all paths from executive functions to physical activity could be constrained to zero across all time points; ‘EF × group → PA’ tested equality of all paths from executive functions to physical activity between intervention groups over all time points. Under the heading ‘two groups’ intervention groups were used in a two-group path model, and under ‘one group’ data were pooled. χ2 = Wald chi-square test statistic, df = degrees of freedom. A significant chi-square statistic indicates that the constraint in question would lead to significant worsening of model fit and to oversimplification.

Joint Stroop, TMT, LF: Joint effects of Stroop, TMT B-A and Letter Fluency tests.

Stroop: Stroop incongruent–Stroop congruent.

TMT: Trail Making Test B–Trail Making Test A.

LF: Letter Fluency.

Baseline performance in any executive functioning test was not associated with baseline physical activity (Table 5). In the pooled data, better baseline Stroop performance predicted likelihood to select higher physical activity response option (i.e. smaller probability to belong to lower physical activity category) in all subsequent time points from six months to COVID-19 (p = 0.015 to 0.030). Better baseline TMT B–A performance predicted the likelihood to select higher physical activity category at six months of the intervention and during COVID-19 (p = 0.006 and p = 0.030, respectively). For Stroop and TMT B–A, changes in executive functions from baseline to six and/or twelve months were not predictive of physical activity at any time point, even though there was a trend towards greater changes in Stroop performance from baseline to six months being predictive of selecting a higher physical activity category at six and twelve months and one year post-intervention (p = 0.087–0.089).

**Table 5:** The effects of executive functioning test performance at baseline and changes in test performance on physical activity from the longitudinal linear path model in full study sample.

	PA_0_			PA_6_			PA_12_			PA_FU_			PA_COVID-19_		
	Est.	S.E.	p	Est.	S.E.	p	Est.	S.E.	p	Est.	S.E.	p	Est.	S.E.	p
**Pooled data**															
Stroop															
Stroop_0_	0.000	0.004	0.977	0.011	0.005	**0.030**	0.013	0.005	**0.015**	0.013	0.006	**0.022**	0.013	0.006	**0.023**
Stroop_6-0_				0.011	0.006	0.087	0.012	0.007	0.087	0.012	0.007	0.089	0.010	0.007	0.172
Stroop_12-0_							0.006	0.008	0.420	0.006	0.008	0.493	0.003	0.009	0.738
TMT															
MT_0_	0.001	0.002	0.420	0.007	0.002	**0.006**	0.003	0.002	0.191	0.003	0.002	0.182	0.005	0.002	**0.030**
TMT_6-0_				0.001	0.003	0.727	-0.002	0.003	0.633	-0.002	0.004	0.621	0.003	0.004	0.385
TMT_12-0_							-0.001	0.003	0.805	0.003	0.003	0.431	-0.002	0.003	0.599
**Two-group model**															
LF, PTCT															
LF_0_	0.013	0.011	0.211	-0.011	0.012	0.358	-0.005	0.012	0.708	0.001	0.012	0.963	-0.009	0.013	0.497
LF_6-0_				-0.007	0.018	0.699	-0.027	0.020	0.173	-0.022	0.021	0.291	0.001	0.021	0.961
LF_12_-_0_							0.015	0.019	0.428	0.010	0.020	0.618	-0.003	0.020	0.874
LF, PT															
LF_0_	-0.010	0.010	0.345	-0.019	0.013	0.127	-0.028	0.013	**0.030**	-0.042	0.013	**0.002**	-0.020	0.013	0.126
LF_6-0_				0.038	0.024	0.111	0.040	0.028	0.148	0.006	0.026	0.826	0.002	0.027	0.946
LF_12_-_0_							-0.054	0.023	**0.020**	-0.006	0.022	0.785	0.007	0.023	0.741

Note.

PA_0_: Physical activity at baseline.

PA_6_: Physical activity at six months of the interventions.

PA_12_: Physical activity at twelve months of the interventions.

PA_FU_: Physical activity at one-year follow up.

PA_COVID-19_: Physical activity during COVID-19 restrictions.

EF_0_: Performance in the executive functioning test in question at baseline.

EF_6-0_: Change in performance in the executive functioning test in question from baseline to six months.

EF_12-0_: Change in performance in the executive functioning test in question from baseline to twelve months.

Stroop: Stroop incongruent–Stroop congruent.

TMT: Trail Making Test B–Trail Making Test A.

LF: Letter Fluency.

For the Letter Fluency test, statistically significant effects were observed only for the PT group in the two-group model (Table 5). Better baseline performance predicted higher physical activity at twelve months of the interventions and during one-year post-intervention follow-up in the PT only group (p = 0.030 and p = 0.002, respectively). In addition, greater improvement in the Letter Fluency test performance from baseline to twelve months predicted higher physical activity at twelve months in the PT group (p = 0.020).

## Discussion

In this exploratory analysis we found that cognitive training, targeting executive functions, in addition to multicomponent physical training did not lead to greater improvements in self-reported physical activity compared to physical training alone among older adults, who did not meet physical activity recommendations prior to the intervention. In comparison to their baseline physical activity, the participants were likely to select their highest activity category after the first six months of the interventions. Remarkably, in both the one-year post-intervention follow-up and extended follow-up during the COVID-19 restrictions, the participants consistently reported a physical activity category higher than at baseline. Our findings also suggest that higher executive function scores at the baseline may predict better adoption to and maintenance of physical activity due to a multicomponent training intervention.

A recent meta-analysis suggested that combined physical and cognitive training interventions lead to greater improvements in executive functions than physical training alone [20], and our previous study lends support to this finding [21]. Higher executive functioning, in turn, is suggested to support healthy behavior such as physical activity [16]. We therefore expected that combining executive functions training with physical training would increase physical activity more than physical training alone, especially after the interventions when continuous supervision and support from the study personnel were ended. However, we did not observe differences between the study groups. One explanation may be, as suggested by Hall and Marteau [22], that the transfer effects of targeted executive functions training remain ambiguous. Thus, the cognitive training of the present study may not have provided sufficient transferable effects to promote healthier behavior in everyday life over the multicomponent physical training.

Adherence to the physical training program was similar in both study groups [21], and the multimodal physical training program itself may have been effective enough to promote adoption to physically active lifestyle and to overcome the impact from cognitive training. As expected based on previous studies [7], the likelihood to report a high physical activity category was attenuated after the end of the interventions in both study groups. However, it is of importance to note that the selected physical activity category of approximately half of the participants was higher than at baseline during the post-intervention follow-up period. Very few previous studies have reported long-term maintenance of physical activity following exercise interventions among older adults, and in those studies the long-term effects have mostly been small or non-existing [7]. One explanation to the relatively good maintenance of physical activity in the present study may be the multicomponent physical training program, which included not only intense supervised training but also home-based exercise to promote adaption to self-motivated training. In addition, behavior change strategies that have previously been considered effective on increasing physical activity [44] were utilized, including feedback and self-monitoring [21]. These intervention strategies seem to have been relatively successful to promote adoption to and maintenance of physically active lifestyle, even though the positive effects slightly attenuated after the first six months of the intervention.

Interestingly, distribution of responses to the physical activity item during the COVID-19 was comparable to the end of the interventions, i.e., even higher than during the one-year post-intervention follow-up. Most previous studies have shown decreased physical activity during COVID-19 restrictions [45, 46], but some have shown increased exercise frequency [47, 48]. Our study is, however, unique compared to other studies on the topic that have mostly been conducted as cross-sectional online surveys [45]. All participants in the present study had received home-based exercise instructions and elastic resistance bands during the interventions, and were thus used to train at home. This may have helped maintain and even increase physical activity during the lockdown. In Finland, older adults also had many possibilities for physical activity outside their homes during the COVID-19 restrictions. Even though people over 70 years were instructed to self-quarantine, no curfew was imposed. In the study area, walkways are good and the nature is close, which create good opportunities for outdoor recreation. In addition, while public sports facilities were closed and training groups were quitted, many private sports facilities remained open. It is also noteworthy that the COVID-19 questionnaire was conducted during April–June, which is an opportune time for gardening and other outdoor activities that are popular among Finnish older adults. In Finland, incidental exercise, and habitual physical activity, such as taking care of errands by foot, is more common than in many other countries, in which physical activity consists mostly of structured exercise. Therefore, it may have been easier for older adults to maintain physically active during the pandemic in Finland than in many other countries.

Even though complementing physical training with executive functions training did not promote physical activity more than physical training alone, we found that higher baseline executive functions predicted selection of higher physical activity category both during and after the interventions. This is in accordance with the conclusion of Greendale and colleagues [49], who suggested that cognitive functioning may impact physical activity in older age and not vice versa. Our finding also lends support to previous studies, which have indicated that executive functions are positively associated with exercise adherence and physical activity [11, 13, 14]. Interestingly, performance in the three executive functioning tests differed in their capability to predict physical activity. This mirrors the Unity/Diversity model of executive functioning–the facets have something in common, yet something different [40]. This may also explain, why the joint effects of executive functions on predicting physical activity did not quite reach statistical significance, but the effects of separate tests were statistically significant–performances in the three tests may be correlated.

Of the three tests utilized, the Stroop was the best predictor of physical activity. Baseline Stroop performance could predict physical activity throughout the study, and there was also a trend towards greater changes in Stroop performance during the first half of the interventions to predict greater probability to select a higher physical activity category in subsequent measurements. This is reasonable, since this kind of test capitalizes not only on automatic response inhibition, but also on common executive functioning, i.e. the capability to maintain and manage goals and to retrieve and implement the right goals at the right time [40]. Especially during such exceptional circumstances as the COVID-19 pandemic, different kinds of goal-setting and goal-oriented behavior are required to independently engage in a physically active lifestyle than to follow a structured and supervised exercise program.

In contrast, the TMT B–A test capitalizes on the shifting facet of executive functioning, which is characterized by requirement of rapid switching between goals [40] and may thus be more essential for adapting to novel situations than for long-term maintenance of physically active lifestyle. It is therefore understandable, that TMT B–A performance may have reflected on physical activity participation at the beginning of the interventions and during the COVID-19, when rapid adaptation to new ways to act was required. Interestingly, better performance in the Letter Fluency test, which is a measure of working memory updating [40, 41], predicted higher physical activity only in the physical training alone group. It may be that the cognitive training in addition to physical training challenged working memory more than physical training alone, and thus the baseline Letter Fluency performance had less predictive effect in the combined training group, even though no between-group differences were observed in test performance [21].

All in all, our findings support the previous evidence that has suggested a positive, bidirectional relationship between executive functions and physical activity, but more research is required on the topic. Future research is needed in more diverse study populations to confirm our findings, and with larger sample sizes to investigate the joint effects of executive functions on physical activity. The initial physical activity category selected by our participants was relatively homogenous in accordance with the aims of the PASSWORD-study, which may be one reason to why we did not observe any associations between executive functions and baseline physical activity. More research is thus required to investigate if executive functions play a role in adoption to physical activity among e.g., more active older adults and those who have contraindications to intense exercising. Investigating the joint effect of executive functions on adoption to and maintenance of a physically active lifestyle in a larger sample would be important to reach sufficient statistical power. It would also be fruitful to investigate, if simultaneous training of executive functions and physical exercise, i.e., doing both during the same session, would promote physical activity more than physical training alone, as recent meta-analyses suggest that simultaneous training has greater effects on cognition than doing cognitive and physical training separately [20, 50].

### Limitations

This study has several limitations. First of all, this study was an exploratory post-hoc analysis of a randomized controlled trial. Thus, power consideration was not extended to the outcomes of the present study. Exploratory analyses are hypothesis generating in nature, which in the present study denotes that the goals of the study were generated, and analysis plan designed after the data collection according to the original research plan of the PASSWORD-study was almost finalized, and an additional data collection was conducted during the COVID-19 pandemic. Additionally, the endpoint (i.e., self-reported physical activity) was re-categorized after inspecting the data. For these issues, a minimally adequate sample size was not possible to be determined by conducting a power analysis. However, the exploratory approach can be considered as a strength in the present study since majority of COVID-19 related research is cross-sectional and thus lacking comparison data from the time before the pandemic.

Second, physical activity was measured using a single self-report questionnaire item. Self-reports are based on questionnaire items with a limited range of options, which restricts response information content and, hence, power, with an impact on the ability to detect associations. Also, it is difficult to assess inter-subject comparability of activity category selection, i.e., whether the participants perceive the response options to be equidistant and if the options represent the same kind of real-life activity participation. Device-based measurement of physical activity or a more detailed physical activity questionnaire could have provided activity data with a wider range of variability, but such activity measurement tends to have low repeatability. Self-reported activity level tends to vary less in repeated measurements and may, thus, yield more stable estimates of long-term activity than highly varying device-based assessments. Additionally, attrition analyses showed that participants who dropped out were slightly older, less active, and less fit than participants who remained in the study. It may thus be that the proportion of subjects choosing high physical activity categories during and after the interventions give an over-optimistic picture of the development of physical activity participation. However, drop-out rate was relatively low throughout the measurement time points. Finally, the results of this study are not likely to be generalizable to those older adults, who were not eligible for the present study. We do not, for example, know if older adults with cognitive decline or disabled physical function would benefit from a multimodal training program. Furthermore, differences in exercise and physical activity culture may restrict generalizability of the results outside Finland.

## Conclusions

Cognitive training targeting executive functions in addition to a yearlong physical training did not lead to greater increase in physical activity than physical training alone among relatively healthy older adults, who did not meet physical activity recommendations prior to the study. Participants in both study groups were likely to report higher physical activity through all subsequent measurements from six months of the interventions to the time of COVID-19 lockdown than at baseline. It may be that the intensive yearlong multimodal physical training program, including not only supervised but also home-based exercise, was effective enough to support adaptation to a physically active lifestyle. Higher baseline executive functions predicted higher physical activity during and after the interventions, even though the predictive effect varied somewhat according to the test utilized and, for letter fluency, according to the study group. Promoting executive functions may be one additional valuable tool in fighting against physical inactivity pandemic among older adults.

## Supporting information

S1 File CONSORT checklist. (PDF)

S2 File Research plan. (PDF)

S1 Table The joint effects of executive functions on physical activity in pooled data. (PDF)
