# Supplementary material for: The effects of a physical and cognitive training intervention vs. physical training alone on older adults’ physical activity: A randomized controlled trial with extended follow-up during COVID-19
Source: PLoS One. 2021 Oct 13;16(10):e0258559. doi: 10.1371/journal.pone.0258559 (PMC8513828; doi:10.1371/journal.pone.0258559)
Supplement: S2 File — (PDF) [file pone.0258559.s002.pdf]

RESEARCH PLAN

Translation of the original foreword (in Finnish) to the ethics committee:

This is the original research plan, which was submitted to the Academy of Finland. Total budget of the research project is 579 243 €, but the Academy of Finland granted the research only 500 000 €. Additional funding is applied from foundations and ministries. Because complementary funding has not been secured yet, resources required for the study have been adjusted to correspond the Academy of Finland grant. The main adjustment is to leave out three months measurements. Furthermore, the research group has grown with a few persons after the funding decision. We have also decided to ask permission from the participants to use register data about the use of social and health services (register of the Finnish Institute for Health and Welfare) and medication (register of Kela (National Pension Institute)) in this study. PASSWORD BRAIN part study will include MEG-measurement without EEG.

Additional information regarding the present study: Extended follow-up during COVID-19

An additional follow-up questionnaire was conducted during the COVID-19. For that, ethical approval was received separately. When applying for the ethical approval, the original research plan was attached, with the above-mentioned original foreword in Finnish.

The research plan below was originally written in English.

1. PROJECT TITLE, PRINCIPAL INVESTIGATOR AND SITE OF RESEARCH

Promoting safe walking among older people: Physical and cognitive training intervention among older community-dwelling sedentary men and women (PASSWORD).

Sarianna Sipilä. Gerontology Research Center (GEREC), Department of Health Sciences, University of Jyväskylä (UJ). Date 29.9.2015.

2. RATIONALE

A major task facing aging societies is to develop effective strategies to promote and improve older people's functional capacity and participation in society. Among the most important factors is the ability to walk safely and independently in one's environment. Safe walking facilitates a physically and socially active life and access to goods and services[1].

In a population based survey conducted in Finland[2], one-half of women and one-third of men 75 years and older perceive difficulties in walking 500 meters. Moreover, approximately 60% of women and one-third of men are unable to walk fast enough (>1.2 m/s) to cross the road during the green traffic light. Walking limitation and disability are associated with poor health, restricted participation in society[3] and falls[4], the majority of which occur while walking. Among the Finnish population, a fall is the most prevalent accident with fall-related injuries accounting annually for 14 000 hospital inpatient periods. The costs of treating fall-related injuries account for 0.85% to 1.5% of total health care cost in the US, UK and EU[5] and 6% of all medical costs for persons aged 65 and older[6].

Safe and stable walking is a complex process involving the interaction of neuromuscular, sensory and cognitive functions[7,8]. The physiological prerequisites for walking are lower body muscle strength and power, postural balance and endurance[9]. Of the higher-order cognitive functions, executive functions correlate with walking ability and predict falls among community-dwelling older people[8,10,11]. Executive functions are cortical processes, which integrate and modify information from several brain regions and contribute to effective, flexible and goal directed behaviour[12,13].

Physical and cognitive functions deteriorate with ageing. Therefore, activities requiring simultaneous physical and cognitive function are especially challenging for older people[14]. A cognitive challenge encountered simultaneously when walking, decreases walking speed and increases variation in the walking parameters in older people[8,10]. It has also been shown that in challenging walking conditions older people

prioritize walking and postural stability over cognitive tasks in order to avoid stumbling and falling[15]. This suggests that to avoid adverse outcomes older people prioritize the sensorimotor task over the cognitive task[15]. Consequently, an elderly pedestrian may be unable to distinguish relevant stimuli from a large array of irrelevant stimuli while walking outdoors or to integrate all the incoming information needed to plan an appropriate route. A recent study using a simulated street-crossing test showed that among healthy older people slow walking and processing speed, poor visual attention and attention shifting were independent predictors for dangerous crossing strategy[7]. In Finland, half of the fatal pedestrian accidents (years 2000-2006) involved people aged 65 years and older.

Physical activity is most likely a key factor in promoting safe walking in older people. To benefit health and well-being across a broad spectrum, the current physical activity guidelines comprise a diverse range of physical activities to be performed on a weekly basis. A recently published large scale trial (The LIFE -study) showed that supervised moderate intensity training, including walking, strengthening and balance exercises, improved walking speed and physical performance but not cognitive function among 70- to 89-year old participants at risk for disability[16,17]. In another study, resistance training intervention resulted in significant improvements in muscle power but not in walking speed among 65- to 75-year old women compared to a balance and toning control group[18]. Interestingly, in the latter study significant improvements by training were observed in cognition, especially in executive functions. They also showed that greater improvement in executive functions was associated with better maintenance of physical activity over a one-year follow-up. Moreover, a recent falls prevention program including supervised strengthening, balance and functional exercises improved muscle strength and mobility in some but not in all tests in 70- to 80-year-old women with a history of falls[19]. The intervention had no effects on the overall rate of falls, but it reduced the rate of injurious falls and injured fallers.

Research on the effects of cognitive training on walking among older people is scanty. Two pilot studies showed that a computer-based program targeting executive functions improved walking speed among healthy older people[20,21]. These findings indicate that cognitive training may have effects on an untrained task with a large neuromuscular component. The exact underlying mechanisms remain unclear, but evidence from elsewhere show that transfer may occur if the training and the transfer tasks share common processes and involve the same brain areas[22]. Earlier studies show that the frontal lobe, especially the dorsolateral prefrontal cortex is activated by both walking and executive functioning[8,12].

In sum, the current scientific evidence on the effects of physical training on safe walking and falls prevention is encouraging but partly conflicting. Therefore, new strategies are needed to promote functional capacity of older populations. The interplay between higher cognitive functions and walking suggest that not only physical but also cognitive training has potential benefits for the prevention of mobility limitation and disability in older people. To date, only a few experimental studies have investigated the effects of cognitive training on walking among older people. In those studies, the training regimens didn't follow any existing guidelines or evidence-based regimens and the studies have been small-scale. Moreover, these studies have been unable to detect possible additive effects of cognitive and physical training.

Link to previous research by the research group: This study is a logical continuation to our research on the promotion of functional capacity among older populations through physical activity. Prof. Sipilä has led studies investigating the effects of physical activity and rehabilitation on functional capacity and mobility among clinical and sedentary older people. We have shown that older people can participate safely in (and benefit from) progressive physical training programs[23-29]. We have also shown that training interventions show greatest benefits in trained task whereas gains in untrained task are less evident and that the benefits of the intervention are maintained in the trained activities, but not necessary in the untrained ones[24,27]. Our recent findings suggest that while physical training is beneficial, other factors such as pain, fear of falling and psychological factors may interfere with mobility recovery and adaptation to physical activities in older people recovering from a hip fracture[30,31]. Specifically, we found that weaker sense of coherence, a measure of how people view life and maintain their health in stressful situations, was associated with poor adherence in organized training sessions and that among this group training did not improve performance in complicated walking tests even after controlling for training adherence[31].

To take a step forward, we have designed a study which investigates the potential additive benefits of two proven interventions, physical and cognitive training, on walking speed and falls among older

sedentary people (Figure 1). Both interventions rely on evidence based findings and they have been tested in large scale trials (the FINGER -study and the LIFE -study) with good compliance and results[16,32].

Modification of the research plan submitted last year: Although the expert panel of the Academy of Finland did not grant funding for this plan last year (overall rating 4), they nevertheless concluded that "This is a novel and timely application that has real potential for a rapid implementation that could both reduce the risk of falling in older people and give insight into the mechanism underlying physical and cognitive training and interaction between them". The criticism pointed out by the panel was related to the study design with four research arms: physical training (PT), cognitive training (CT), physical& cognitive training (PTCT) and a health education control. The panel recommended that the design should be simplified into a two arm trial with PT and PTCT groups only. They also suggested that the primary outcome should be walking speed over 10 m instead of walk distance over 6 min. After giving careful consideration to the panel's suggestion, we decided to revise the application accordingly; hence the study design and the primary outcome have been changed, the research questions rewritten and the sample size recalculated.

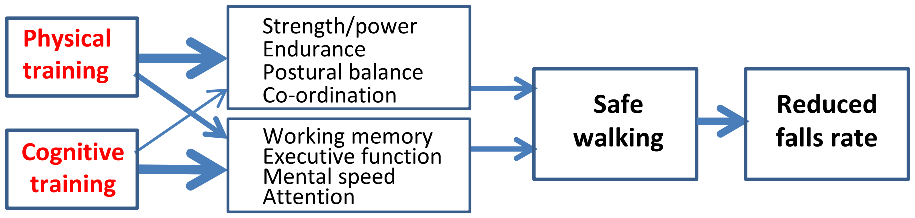

Figure 1. Conceptual model of the study. The thickness of the arrows indicate current scientific evidence.

3. OBJECTIVES AND EXPECTED RESULTS

Objectives and hypotheses of the study. The overall objective of this study is to investigate, in 70-85-year-old community-dwelling sedentary men and women, whether the combination of physical and cognitive training has greater effects on walking speed than physical training only. The effects of the interventions on falls will also be investigated. The specific aims, hypotheses and justifications for the hypotheses are the following:

MAIN AIM: To investigate, in older community-dwelling sedentary men and women, the effects of a 12-month structured progressive physical and cognitive training program (PTCT) on walking speed and dual-task cost in walking speed compared to physical activity (PT) only.

Hypothesis: The PTCT will induce significantly greater benefits on walking speed than the PT only. Dual-task cost is smaller after the PTCT intervention than after the PT-only intervention.

Justification: The key determinants of safe walking (physical and cognitive function) are amenable to physical activity interventions even in old age[24,25,29,33,34]. Cognitive training has beneficial effects on cognition and there is suggestive evidence that cognitive training may have beneficial effects on walking speed among older people[20,21]. A training program targeting both the key determinants underlying safe walking is likely to induce greater benefits than a program targeting only one key determinant.

SECONDARY AIM: To investigate the effects of the PTCT program on rate of falls during 12-month intervention and a one-year follow-up in the target population compared to the PT only.

Hypothesis: The rate of falls will be smaller among the PTCT group than the PT group during the intervention and a one-year follow-up.

Justification: Epidemiological studies show that a higher level of physical activity and greater cognitive ability are associated with reduced fall risk[11]. Although there are no consistent findings showing that physical training interventions reduce falls or, in the absence of well-designed trials, any direct evidence that cognitive training has similar benefits, it is reasonable to speculate that training in these two key factors would have additive effects and thus result in a significant reduction in the rate of falls. The effects of training interventions wane overtime if participants return to the sedentary behaviour following intervention cessation[27,35]. A recently published physical activity intervention showed that improvement in executive functions during a trial was associated with better maintenance of physical activity over a one-year post-intervention follow-up[35]. In our study, greater improvements in executive function are expected in the PTCT compared to the PT, as executive functioning is a key target of the CT. Maintenance of the

physical activity (and potentially cognitive training) over the follow-up will have a protective influence on falls.

THE BRAIN SUB-STUDY: Brain maintains the ability to adapt to changes in physical and cognitive (mental) load up to the old age. Aerobic exercises, such as walking, increases efficiency in the brains information processing, especially in networks related to executive control, and activation of the sensorimotor network[36]. Resistance training has also been shown to induce functional changes in cortical regions which are associated with response inhibition processing during executive functioning[34]. In older people, cognitive training induces task-specific behavioural changes together with related adaptation in brain activity[22]. Knowledge on the additive effects of physical and cognitive training on brain function is, however, scarce. In collaboration with the Center for Interdisciplinary Brain Research at the UJ (CIBR) we will investigate the combined effects of PTCT on attention-specific sensory and cognitive processes in the brain by utilizing simultaneous electroencephalography (EEG) and magnetoencephalography (MEG) recordings. The essential parts of attention, arousal, orienting and executive functions are relatively independent factors. The sensory (bottom-up) component of selective attention deteriorates gradually by aging, which might be inevitable. In contrast, the top-down control of selective attention might be alleviated by the interventions of this study.

Potential for scientific breakthroughs and for the regeneration of science and research. The current study will provide new knowledge on the effects of physical and cognitive training on walking and falls prevention in older sedentary people. This study is designed to capture additive effects of physical and cognitive training by using evidence-based guidelines and training regimens and a comprehensive battery of validated walking tests. The interventions will be specifically designed to facilitate the adaptation to active lifestyle. Expected results may lead to a breakthrough in research and strategies aiming to promote safe walking and functional capacity of older populations.

Expected scientific and societal impacts, applicability and utilization potential of research results. The project will provide new insights for the promotion of safe walking by increasing our understanding of the importance of physical and cognitive training among older sedentary people. The results may form basis for future studies which aim to investigate e.g. rehabilitation strategies among older disabled and clinical populations. The brain sub-study will provide important pilot data for the future mechanistic studies. The results may significantly contribute to the national and international guidelines and rehabilitation strategies aiming to promoting health and functional capacity of older populations. The expected results may also have a significant socio-economic impact. This research project will educate interdisciplinary experts and researchers in the field of gerontology able to develop and test sustainable strategies for the benefit of aging societies.

Critical points for success and alternative implementation strategies. *Recruitment:* Nine percent of the inhabitants of the city of Jyväskylä are aged 70-85 (n=12 238). The population should be large enough for this study (n=310). In our previous studies, 30% (in clinical populations) to 60% of the older citizens meeting the inclusion criteria have attended our studies with a dropout rate below 10%[28,37]. We consider the risk of not being able to recruit participants during the planned time period minor one.

Physical *exercise interventions and maximal performance testing:* Use of walking poles during walking exercises will be recommended, as such exercises could increase fall rates in older people. Falls will be monitored on a monthly basis with diaries. If a significant increase in fall incidence is observed, interventions will be modified after close analysis of the falls data. No major health concerns have arisen in our previous studies[28]. First aid will be available together with examination by a nurse and/or physician when needed. All participants and personnel will be covered by insurance taken out by the University.

Contamination of study groups: All participants will receive the same physical training intervention and half will also receive cognitive training. PT-only group may become interested in cognitively challenging exercises. They are, however, unlikely to adapt a training regimen as intensive as that designed for this study and also organized separately. Some studies have used cross-over and cluster randomization to overcome the risk of contamination. In this study, due to the post-intervention follow-up, it is crucial that all participants remain in their original randomly assigned study group. Moreover, the effects of the physical and cognitive training may last for months[27,38] which in this study would mean an over-long washout period. Cluster randomization designs are susceptible to recruitment bias and require much larger sample size than individually randomized trials[39].

Adherence and drop-out rates: Drop-out was taken into account in our sample size calculations. Adherence will be monitored and drop-outs carefully documented. The effects of the intervention will be analysed by the intention-to-treat –principle.

Double blinding: Double blinding is not possible. To ensure blinding of the study personnel, participants will be asked not to disclose their group allocation to the assessors or other participants.

Personnel: It is important that the *personnel* involved in the data collection remain unchanged in repeated measures designs. Our experience is that research assistants recruited from among University students are able to commit to long-term research projects. We will, however, have a back-up system for data collection in place in case of sickness leaves and other impediments.

Sample size calculations: No data on the combined effects of PT and CT were available. We were able to find reliable data on the effects of PT on walking speed, but data on the effects of CT were scanty. Only a few small-scale pilot studies on the effects of CT have been published and in those studies a large treatment effect (>10%) was observed. Due to the small amount of available data and possible publication bias related to the effects of CT on walking speed, we have halved the earlier published treatment effects for the sample size calculations (for details, see paragraph 4).

Publication plan. The results of this study will be presented in national (Gerontology Meeting 2018 and 2020; Annual Sports Medicine Meeting 2019-2020) and international scientific meetings (GSA and ACSM 2019, 2020). We target the highest ranking scientific journals in Gerontology and Geriatrics prioritizing open access series with green or golden open access policy. We will promote the visibility and usability of the research results through the Digital Archive of the University of Jyväskylä (JYX). JYX, an institutional repository providing access to content created by staff members (e.g. by parallel publishing, http://openaccess.jyu.fi/en/ welcome) managed by the University library. Press releases will be issued on all the published results. The results will also be presented in meetings and written for journals intended for health care and physical activity professionals and general public.

This study collaborates with key stakeholders able to transfer knowledge into practice: **1**) Two Universities of Applied Sciences, which educates health care professionals, **2)** the Department of Sports and Leisure of the City of Jyväskylä, which organizes physical activities for older citizen, **3**) the University of the Third Age (UTA), which organizes lectures and seminars for older citizen on a weekly basis, **4)** the Research and development foundation GeroCenter, which aims to promote functional capacity and wellbeing among older people (PI is the chair of the board of directors).

4. RESEARCH METHODS AND MATERIAL, SUPPORT FROM RESEARCH ENVIRONMENT

Study design. *This study is a parallel group single blinded randomized controlled trial (RCT) with two research arms; Physical Training (PT, control) and a combination of Physical and Cognitive Training (PTCT).* The use of randomization and pre- and post-intervention measurements ensure the internal validity of the study and enable causal conclusions between the interventions and the main outcome[40]. The design controls for confounding factors present before the study (e.g. age and general health of the participants) and those occurring during the study (e.g. change in season, adaptation to the measurements and aging). The interventions will last for 12 months. Measurements will be organized at baseline and at 6 and 12 months. Falls data will be collected during interventions and during the one-year follow-up. The study protocol has been designed according to the current guidelines for RCTs and will be registered in the International Standard Randomized Controlled Trial Number Register (ISRCTNR).

Participants and recruitment. Community-dwelling sedentary 70- to 85-year-old men and women will be recruited from the Jyväskylä population register. Jyväskylä is the seventh largest city in Finland and its population is comparable to the general Finnish population. In 2014, 9 % (n=12 238) of all the inhabitants were 70-85-years-old. In other comparable sized cities in Finland, the percentage of 70- to 85-year-old inhabitants is ~10 %.

Participants will be recruited in two waves (WAVE1, n=155 and WAVE2, n=155 in **Table 3**) to overcome the challenges related to the measurement and training of 310 participants. In both WAVEs, recruitment will start with an information letter sent to a randomly selected 4000 (1/3 of the population) persons. The letter will contain information about the study and the announcement to expect a phone call during the following week. Phone numbers will be collected from a nationwide database. Repeat phone

calls will be made if necessary. After the telephone interview, examination by a nurse, and if necessary, a physician, will be arranged. *Inclusion criteria* are **1**) age 70 to 85, **2**) living independently in the City of Jyväskylä, **3**) sedentary or only moderately physically active (less than 150 min of walking per week and no attendance at gym activities), **4**) able to walk 500 m without assistance, **5**) intact cognition (Mini Mental State Examination Score, MMSE ≥24), and **6**) informed consent to participate. *Exclusion criteria* are **1**) severe chronic condition or medication affecting cognitive and/or physical function (e.g. cancer, severe psychiatric disorder, **2**) excessive and regular use of alcohol (more than 14 portion per week in women and 21 in men), **3**) contraindication for physical exercise or walking tests[41].

If, for some reason, we are unable to recruit enough participants with this strategy, we will send the information letter to the remaining one third of the population that was not contacted during the initial recruitment phases.

Randomization and blinding. Participants will be randomized into groups of equal size after baseline measurements. A computer-generated random allocation sequence of two-fold stratification by gender and age (70-74, 75-79, 80-85) with blocks of 20 will be designed by a biostatistician blinded to the study participants. After the baseline, the research coordinator will run the randomization program to assign the participants to the study groups. Group allocation will only be known by the research coordinator, nurse and physician. Outcome assessors, but not participants, will be blinded to study group.

Sample size. A priori sample size calculations are based on previously published and our own data on 10-m walking speed[19-21]. Our own data (SCAMOB)[42] give reason to expect a baseline mean level of 1.3 m/s (standard deviation of 0.36 m/s) in both groups. We expect the PT intervention to induce a 4 percentage point mean increase in both groups and a 6 percentage point higher mean in the PTCT group than PT group. The SD is expected to remain unchanged in both groups. The follow-up within-person correlation for the two measurements, obtained from SCAMOB, is estimated to be r=0.80, yielding 0.23 m/s as an estimate of the SD for change. Setting the significance level at 0.05 and power at 80 % for the group-time interaction favouring the PTCT vs. the PT group indicated that a sample size of 135 participants per group would be required. The power analysis was based on Diggle et al. (2002)[43] and verified by a simulation study with Mplus-software version 7. Given an anticipated dropout level of 15 %, we decided to recruit 155 participants per group. A additional power analysis was calculated for the secondary outcome of falls rate based on recently published data[19] and formulae in Zhu & Lakkis (2014)[44] for negative binomial distribution outcomes (intercept: 0.56, dispersion 0.64). At 80 % power and with a total sample size of 270-310, depending on the dropout rate, it would be possible to detect a difference of 27-29 % in falls rate significant (α= 0.05) between the PT and PTCT groups.

Our sample size calculations are conservative. For comparison, a recent meta-analysis reported the mean effect of multicomponent PT on walking speed to be 8%[45]. The mean effect of CT on walking speed has been reported to be 11%[20]. Moreover, in a recent two-year falls prevention study, a 30% difference in falls rate between the exercise and non-exercise control group was expected[19].

Measurements. *The main outcome of this study is 10-m walking speed.* Participants are asked to walk as fast as possible over the 10-m course. The time to complete the walk will be measured by photocells. Low walking speed is associated with e.g. increased risk for disability, cognitive impairment, institutionalization and falls[46]. It has also been used to establish thresholds for community activities such as the ability to cross the street safely.

Secondary outcomes are 6-min walking distance, dual-task cost in walking speed and fall incidence. In the 6-min walking test participants are encouraged to walk up and down a 20-m circuit for six minutes covering as much distance as possible. No verbal encouragement is provided during the test. Six minute walking test serves as a measure for community walking and it is associated with e.g. mobility limitation and disability[47]. The visuospatial dual-task walking test will be adopted from that used by Menant et al[48]. Participants are asked to walk at self-selected speed along a 20-m long walkway. After that they will be asked to walk the same walkway while performing a visuospatial cognitive task. Walking times will be measured by photocells and the difference between the two walks (dual-task cost) will be calculated. The visuospatial task involve a display with three boxes side by side labelled A, B and C. Participants are asked to visualize a star located in one of the boxes making three movements. Prerecorded instructions will deliver the random starting position and the direction of the three movements, i.e. left or right. The cognitive task instructions will be delivered continuously throughout the walking trial

through headphones. A new instruction will be delivered within a second of the participant answering the previous question. Participants will practice the visuospatial task carefully before the dual-task test. Fall incidence will be monitored by monthly diaries throughout the study. Phone interview will be used to ascertain further details of falls and injuries and to chase missing data as suggested by the Prevention Of Falls Network Europe. A fall is defined as an unexpected event in which the participant comes to rest on the ground, floor or lower level without overwhelming intrinsic or extrinsic cause.

Overall health including depression (Geriatric Depression Scale) and nutritional status (MNA-SF) will be assessed during a medical examination. To ascertain safe participation in the measurements and intervention, contraindications (ACSM guidelines)[41] and acute conditions (blood count, CRP and Hb) will be assessed.

Other measurements include self-reported difficulty in walking outdoors, 500 m and 2 km[28], life space[49] and fall-related self-efficacy (FES-I). Functional balance (Berg scale), lower limb muscle strength and power[28] as well as body composition (DXA) will also be assessed. Cognitive tests include test for global cognitive function (Consortium to Establish a Registry for Alzheimer's Disease, CERAD total score)[50], executive functions (Stroop and Trail Making)[51] and verbal fluency (Verbal Fluency Test)[52]. Sense of coherence will be assessed at baseline[31]. The level of physical activity will be assessed by a validated questionnaire and accelerometer (Gulf Coast Data Concepts LLC, USA). Accelerometer data will be collected for 7 consecutive days after baseline and 6 month measurements and approximately a week before the 12 month measurements. Data from the two first days will be discarded in order to overcome the temporary change in the level of physical activity due to the monitoring.

The brain sub-study: Neural and cognitive functions will be assessed by using Attention Network task[53] combined with EEG and MEG recordings. The recordings will be made before and after the interventions for 20 randomly selected participants per study group. Briefly, the tests consist of preparatory signal, orienting cue, and decision making. Behavioural measures are reaction times and proportion of correct choices. We will also use an audiovisual task with elements of focused attention, inhibition and shifting of attention. Our setup will allow for assessment of the level of performance at each subfunction and their neural correlates. The MEG/EEG data will be analysed as stimulus-locked evoked responses in stages of attention and as ongoing oscillatory brain activity and the induced modulations caused by task requirements. Modelling algorithms allowing the localization of activation sequences and interpretations at the level of underlying brain areas will be utilized.

Interventions. *The interventions, comprising individually targeted progressive supervised training sessions and home exercises, will last for 12 months. Supervised sessions will aim at the maximal training effects whereas home exercises facilitate the adaptation to active lifestyle. Intensity, resistance, difficulty and number of training sessions will be increased during the training period. Training adherence will be carefully monitored.* Supervised sessions will be organized in groups of 10-15 participants. A diary on all home exercises will be kept on a daily basis. During summertime (June-July), extra home exercises will be provided for those who are unable to attend supervised sessions. During the first month of intervention, physical training only will be organized. This arrangement will allow participants to adapt to the training on a weekly basis. The acceptability of the interventions will be assessed by a questionnaire. For the post-intervention follow-up, encouragement to continue physical and cognitive training, but no support, will be given. The interventions are summarized in **Table 1** and descry bed in the text below.

Table 1. Summary of interventions.

Intervention/Months	1	2-4	5-7	8-10	11-12
**Physical training (PT)**					
Supervised sessions, fr/wk	1	2	2	2	2
Activity, duration	GYM: BT+ST	1 GYM: BT+ST<br>1 Nordic walk:20 min	1 GYM: BT+ST<br>1 Nordic walk:30 min	1 GYM: BT+ST<br>1 Nordic walk:30 min	1 GYM: BT+ST<br>1 Nordic walk:40 min
Home exercises, activity, duration	Walk: 60min spread over the week	BT+ST exercises<br>Walk: 60-80min spread over the week	BT+ST exercises<br>Walk: 80-100 min spread over the week	BT+ST exercises<br>Walk:120 min spread over the week	BT+ST exercises<br>Walk: 110-120 min spread over the week
**Cognitive training (CT)**					
Supervised sessions, fr/wk	-	1	1-2	2	2
Effective duration		10-15 min	15 min	15-20 min	15-20 min
Home exercises, fr/week	-	1-2	2	2	2
Duration		10-15 min	15-20 min	15-20 min	15-20 min

BT=Balance Training, ST=Strength/Power Training, BL and ST exercises will be performed during the same training session.

The multicomponent physical training (PT) intervention will be adapted from the physical activity guidelines, our earlier studies[25,29] and the LIFE -study[16]. The PT intervention will include Nordic walking and progressive resistance and balance training. The duration and distance of the walking sessions will be monitored. The target intensity will be 13 (moderate value) on the Borg scale. Each participant will attend once a week in walking session supervised by physiotherapy students from the local University of Applied Sciences. The walking exercises will be performed outdoors. Opportunities for outdoor activities are well developed in Jyväskylä which has numerous built-in sidewalks and walkways. During winter time, the walking exercises can also be organized in a sports hall with 200 m oval track.

Resistance and balance training will take place in two senior gyms owned by the Department of Sports and Leisure of the City Jyväskylä. The gyms are equipped by HUR resistance training machines with SmartCard Software which stores performed workload, sets, reps, and exercise time during each visit (http://www.hur.fi/en). The Department has a long evidence-based tradition of organizing physical training for older people. Training sessions will be organized specifically for the purpose of this study. Each training session will include balance training followed by resistance training aiming to improve strength and power for the lower extremity muscles.

The cognitive computer-based training (CT) will target working memory and different components of executive functions, that are all sensitive to the aging process and are associated with walking. The CT program will be a modification from the FINGER -study[22,32]. Multidomain CT activates larger neuronal networks and is more likely to elicit transfer effects than single domain training. It also enables variation in training sessions, which may increase training compliance. Computer-based exercises enable individually adjusted progression by increasing the level of difficulty. Training sessions, supervised by psychology students, will be organized in the computer class at UJ. During the first four weeks of CT, peer support for the required computer skills will be organized in collaboration with the local University of the Third Age (UTA). UTA-run Geronet-computer courses have a well-developed peer support tutor system. (https://kesayo.jyu.fi/ikaantyvienyliopisto/en). For the home exercises, those who lack access to a computer at home will have possibility to train in one of ten locations provided by the City of Jyväskylä. (libraries, sheltered accommodations, etc). In each location a Geronet tutor will be present each week for 2-3 hours at a time. We can also provide 10 participants with laptop computers for training at home.

Participants in the PT group will receive PT only and thus, they participate in 1 to 2 supervised sessions per week. *Participants in the PTCT* group will receive both training regimens. In order to facilitate participation in the supervised training sessions, supervised walking sessions will follow one of the supervised CT sessions. Consequently, the participants in the PTCT group will attend 2-4 supervised sessions per week on 1-3 different occasions.

Statistical analysis. The effects of the intervention will be assessed on the intention-to-treat principal using linear mixed models for continuous variables and by a general estimation equation for categorical variables. The primary outcome is tested for group-interaction over time using an interaction contrast ($t_{\text{time}\times\text{group}} = \mu_{\text{BL,int}} - \mu_{\text{FU,int}} - \mu_{\text{BL,cont}} + \mu_{\text{FU,cont}}$, t: contrast test statistic, μ: estimated marginal mean, BL: baseline, FU: follow-up, int: PTCT intervention, cont: PT control) in a linear model for longitudinal data accounting for within-person correlation and different variances at the two time-points. Negative binomial regression will be used to estimate the incident rate ratio for falls. The Cox proportional hazard regression model will be used to calculate hazard rates for fallers in both groups with PT as a reference. In addition, individual changes in the main and secondary outcomes observed during the study will be calculated and a reliable change index computed.

Research material, materials management plan and confidentiality of data. A quality assurance system will be set up. This includes a written standard operation procedure as well as periodical meetings and checks for monitoring the intervention and quality of data collection. The personnel performing the measurements and supervising the interventions will be carefully educated. Data will be collected by questionnaires, interviews, health examinations and laboratory tests. Interview-based data will be collected using computer-assisted personal interviewing (SPSS Data Entry Builder soft- ware, SPSS Inc). Each participant will be assigned an identification number. The identification -key will be in the possession of the research coordinator, research nurse and PI during the data collection and thereafter in the possession

of the PI only. All information collected on paper will be locked in a cabinet at GEREC and saved as data files as soon as possible. Data files will be stored on the UJ server and protected by passwords. The research data will be owned by the UJ and managed by the PI or a person specifically appointed for this task. When participating in the project, researchers will be able to use the data for research and teaching while in the University's employ. If necessary, permission to use the data may also be granted to researchers who change their employer. The main results will be published by the research group. Data will also be available to other investigators for additional analysis, as individually agreed with the research group. Information on the study design and outcomes will be available at the ISRCTNR and in a design paper published in an open access journal.

Research infrastructure. *This study will be conducted at the Gerontology Research Center, Department of Health Sciences, Faculty of Sports and Health Sciences, University of Jyväskylä (UJ, GEREC, http://www.gerec.fi/en).* The GEREC has app. 30 staff members including professors, senior researchers, lecturers, post-doctoral and doctoral students. This project has full support from the Department, the Faculty and the UJ. The UJ has set gerontology as an international leading edge strategy. In partnership with the Health and Physical Activity Laboratory of the UJ, GEREC has extensive up-to-date facilities for the assessment of physical functioning. The biochemistry laboratory of the Faculty has up-to-date methodology needed for the haematology analysis in this study.

CIBR (http://cibr.jyu.fi/) provides state-of-the-art infrastructure for performing brain imaging studies, including e.g. MEG laboratory and several EEG laboratories, behavioural and psychophysical test booths, and a dedicated fire-wall protected data server with modern analysis softwares. Combined EEG/MEG recordings are possible in CIBR. CIBR offers well-structured practical training, access protocols and support personnel for designing and setting up a neuroimaging study, as well as for analysis and technical aspects.

5. ETHICAL ISSUES

The study will be performed according to the guidelines of the Finnish Advisory Board on Research Integrity (http://www.tenk.fi/en/frontpage), good scientific practice and current legislation. The study protocol has been designed according to the current guidelines for RCTs. Application for ethical approval will be made to the Jyväskylä Central Hospital Board. An informed consent, explaining the details of the study, possible risks, and permission to use the data for research purposes, participants´ right to decline to participate at any point, anonymity and confidentially of the data will be signed by the subjects before the baseline measurements. ICMJE guidelines will be followed when reporting the research results.

6. IMPLEMENTATION: BUDGET, TIME SCHEDULE, DISTRIBUTION OF WORK

Budget (see Table 2). The salaries of the senior members are covered by their host institutes. The PI will spend 35% and the other senior researchers 5-15% of their working hours on this project. Travel costs include travel of participants to measurements and training, conference trips and research exchanges between the collaborating Universities. Funding will also be applied from the Ministry of Education and different foundations, if necessary. Funding for MEG/EEG recordings will be applied with this application, but funding for data analysis and reporting will be sought elsewhere.

Table 2. Summary of funding applied for.

Purpose / year	2016	2017	2018	2019	2020	Total
Salaries:						
-Coordinator, NN, 28 person months (pm)	3 833	23 695	24 398	4 139		
-Post doc N.N, 31 pm		8 025	33 058	34 048	11 543	
-Statistician, 1.2 pm		613	1 578	1 626		
-Research assistants, NN, 28 pm		23 128	23 813	3 664		
-Research nurse, NN, 12 pm		12 046	12 408			
-Training surveillance and instructors, 16pm		13 671	14 081			
Indirect employee costs	2 108	44 647	60 135	23 939	6 348	
University overhead	5 585	118 275	159 303	63 417	16 818	
Fees for Physician and ICT technician	3 000	5 000	2 000			
Gym fee, materials, publications, MEG/EEG		24 000	27 000			
Travelling expenses	4 500	3 000	2 000	5 000	2 000	
**Grand total**	**19 027**	**276 099**	**359 775**	**135 881**	**36 709**	**827 490**
Funding: Home institution (30 %)	5 708	82 830	107 933	40 764	11 013	248 247
Funding: Academy of Finland (70 %)	13 219	193 269	251 843	95 117	25 696	579 243

Time schedule. For details, see **Table 3**. Briefly, if the decision for funding is positive, recruitment of research personnel and the planning and designing of the interventions will start in spring and continue during autumn 2016. Participants will be recruited in two waves during 2017. Interventions will take place during 2017-2018 and follow-up for falls will continue until 2020.

Table 3. Time schedule

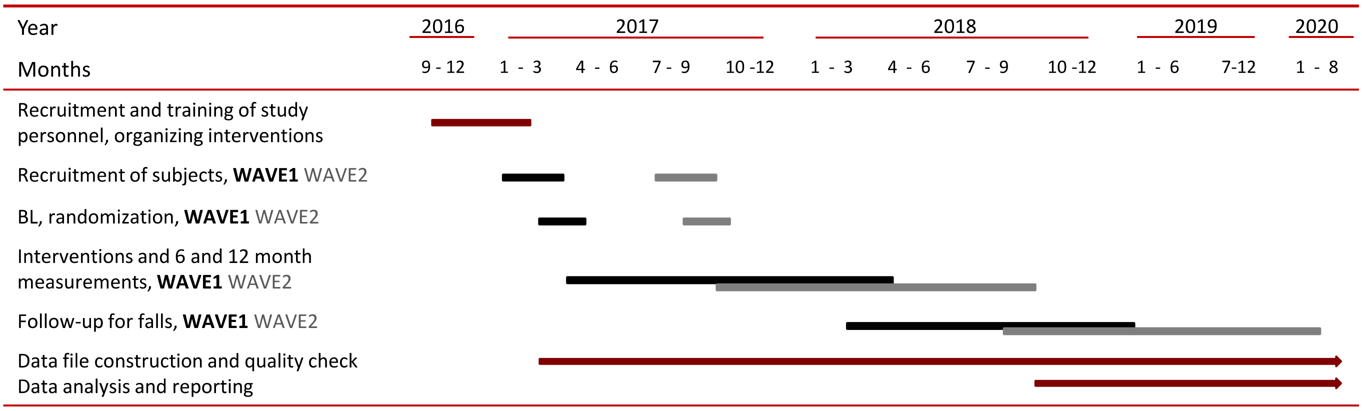

7. RESEARCH TEAM AND COLLABORATION

Professor Sipilä is the principal investigator (PI) of this study. She takes the overall responsibility of this research project and supervision of the post-doctoral and PhD students. She is internationally well-known for her physical activity and rehabilitation studies among clinical and sedentary older people. She has been successful in receiving external research funding as a PI or co-PI with total amount of ~3.3 million euros during the last 10 years. She has been active in research training and is currently a work package leader in an ITN "Physical activity and nutrition influences in aging" funded by the EU H2020. UJ has awarded Prof. Sipilä for excellence in leadership in 2010. Sipilä has been nominated in a number of positions of trust *in scientific communities*, such as Research council for health, the Academy of Finland; Centre for interdisciplinary brain research, UJ*;* JPI More years better life and in positions of trust *in society* such as, Advisory board for public health, Ministry of social affairs and health; the Foundation for research and development GeroCenter; University of the third age, UJ.

The multidisciplinary research team includes expertise in gerontology, geriatrics, sports medicine, sport sciences, cognitive neuroscience, rehabilitation and psychology. The research group and their expertise are summarized in **Table 4** and the division of work in **Table 5**.

Table 4. Research group and their expertise

Name	Expertise
**Alen** Markku, MD, PhD, Professor, Specialist in Sports and Exercise Med.[1]	Long-lasting expertise in clinical and medical issues related to physical activity and health
**Kulmala** Jenni, PhD, Senior lecturer, researcher[2,3]	Sensory and cognitive functions as determinants of functional capacity among older people. Has established collaboration with Prof. Kivipelto
**Rantanen** Taina, PhD, Prof. Gerontology and Public Health[1]	Internationally recognized expert in research on disablement process and how to prevent disability in old age
**Sihvonen** Sanna, PT, PhD Principal Lecturer[4]	Expertise on fall prevention and balance training. Author of the Fall Prevention Guidelines issued by the National Public Health Institute
**Sillanpää** Elina , PhD, Post Doc Researcher[1]	Expertise in physical performance methodology and physical training programs
**Sipilä** Sarianna, PhD Prof. Exercise Gerontology[1]	Physical activity and exercise among healthy and clinical older populations. PI of the study. Member of the Advisory Board of the CIBR
**Törmäkanga**s Timo, PhD,Statistician[1]	Biostatistics with experience in working in longitudinal ageing studies
Post Doc Researcher[1], PhD, N.N	PhD in Gerontology, Biology of Physical Activity or Psychology
Research Coordinator[1], MSc, N.N	Coordination of long-term research projects with repeated measures design
Research Nurse[1], MSc, N.N	Health and clinical examinations among older people

[1]GEREC and Department of Health Science, UJ, [2]Seinäjoki Univ. of Applied Sciences, [3]Chronic Disease Prevention Unit, National Institute for Health and Welfare, Helsinki,[4]Jyväskylä Univ. of Applied Sciences

Table 5. Division of work within the research group

	Method expertise	Recruitment of subjects	Medical screening, surveillance	Data collection	Designing and supervising interventions	Data file construction	Data analysis	Reporting
Alen		x	x	x				x
Kulmala					x	x	x	x
Rantanen	x						x	x
Sihvonen	x				x			x
Sillanpää	x			x	x		x	x
Sipilä	x	x		x	x	x	x	x
Törmäkangas	x						x	x
Post doc				x		x	x	x
Coordinator		x		x		x	x	x
Nurse		x		x	x	x		x
Assistants				x	x	x		x

Collaboration will be build up on acknowledged expertise on physical activity and cognition among older populations. *Miia Kivipelto* is Professor of Clinical Geriatric Epidemiology at the Karolinska Institutet, Stocholm, Sweden. She is internationally recognized for her research on the prevention, early diagnosis and treatment of cognitive impairment, dementia and Alzheimer's disease. She is the principal investigator of the FINGER –study[32]. *Tuomo Hänninen* (PhD, Department of Neurology, Kuopio University Hospital, Finland) is a psychologist recognized for his expertise in the Consortium to Establish a Registry for Alzheimer's Disease (CERAD) neuropsychological test battery. He belongs to the FINGER research group and will have a crucial role in cognitive measurements of this study. *Professor Roger Fielding* is a director of the Nutrition, Exercise Physiology, and Sarcopenia Laboratory at the Tufts University, USA. He is internationally renowned for his research on the impact of physical activity and exercise on successful aging. He is one of the principal investigators of the LIFE -study[16]. He will have a crucial role in developing and modifying the PT program for the purpose of this study. The *brain sub-study* will be performed in collaboration with the CIBR and Department of Psychology at the UJ. The key persons for collaboration are the head of the CIBR *Tiina Parviainen* (PhD), an expert in using MEG to study human brain functions and adjunct professor *Jan Wikgren* (PhD) expert in neurophysiology underlying learning and memory and in use of methods ranging from animal intracranial recordings to human EEG.

8. RESEARCHER TRAINING AND RESEARCH CAREERS

It is aimed to recruit a post-doctoral researcher for this study as well as two new PhD students from among the assisting personnel of this study. Funding for the PhD students will be sought elsewhere. This project promotes gender equality. When recruiting personnel to this project, equal opportunities will be given to all applicants. Supervision of the PhD student and post-doctoral researchers will be carried out by the project PI and senior members of the research group. Doctoral students will be enrolled on postgraduate studies organized at the UJ graduate school. Post-doctoral researcher and PhD students will present their work annually in national and international scientific meetings, thereby learning to enter into dialogue with other scientists. Scientific meetings also enable professional networking, a necessity for future professional success. All the senior members of the research group have a strong commitment to research training. The interdisciplinary group at GEREC offers a creative and successful environment for research training. The PI has supervised nine PhD theses, three with honours and three graded as excellent. In addition, five post-doctoral researchers have worked in her research projects.

9. MOBILITY PLAN

Discussions and exchange of opinions related to details of the study plan will be implemented between the collaborators by e-mails, phone calls and internet platforms. Prior to the beginning of the data collection, the PI and the research assistants responsible for cognitive testing and training will visit Dr. Hänninen at Kuopio University Hospital in order to learn the cognitive testing protocol. In autumn 2016, the PI will visit Prof. Kivipelto at the Karolinska Intitutet in order to finalize the CT program and to consolidate the collaboration. Prof. Fielding will visit GEREC in autumn 2016 for one week. He will have a crucial role in helping to design and finalize the PT program for the purpose of this study. To finalize the plans for joint articles Prof. Sipilä will spend two weeks at the Tufts University in spring 2019. In addition to this, the post-doctoral researcher will visit Prof. Fielding at the Tufts University for six months in 2019.

10. KEY LITTERATURE

1. Satariano WA et al. *Am J Public Health*. 2012;102(8):1508-1515.
2. Koskinen S et al. eds. *Terveys, toimintakyky ja hyvinvointi suomessa 2011*. THL; 2012.
3. Hardy SE et al. *J Gen Intern Med*. 2011;26(2):130-135.
4. Ganz DA et al. *JAMA*. 2007;297(1):77-86.
5. Heinrich S et al. *Osteoporos Int*. 2010;21(6):891-902.
6. Axer H et al. *Clin Neurol Neurosurg*. 2010;112(4):265-274.
7. Dommes A et al. *Accid Anal Prev*. 2013;59:135-143.
8. Yogev-Seligmann G et al. *Mov Disord*. 2008;23(3):329-42; quiz 472.
9. Rantanen T et al. *J Am Geriatr Soc*. 2001;49(1):21-27.
10. Faulkner KA et al. *Gait Posture*. 2006;24(2):182-189.
11. Mirelman A et al. *PLoS One*. 2012;7(6):e40297.
12. Burzynska AZ et al. *Hum Brain Mapp*. 2012;33(7):1607-1620.
13. Fisk JE et al. *J Clin Exp Neuropsychol*. 2004;26(7):874-890.
14. Schaefer S et al. *Gerontology*. 2011;57(3):239-246.
15. Doumas M et al. *Exp Brain Res*. 2008;187(2):275-281.
16. Pahor M et al. *JAMA*. 2014;311(23):2387-2396.
17. Sink KM et al. *JAMA*. 2015;314(8):781-790.
18. Liu-Ambrose T et al. *Arch Intern Med*. 2010;170(2):170-178.
19. Uusi-Rasi K et al. *JAMA Intern Med*. 2015;175(5):703-711.
20. Smith-Ray RL et al. *Health Educ Behav*. 2014;41(1 Suppl):62S-9S.
21. Verghese J et al. *J Gerontol A Biol Sci Med Sci*. 2010;65(12):1338-1343.
22. Dahlin E et al. *Science*. 2008;320(5882):1510-1512.
23. Kallinen M et al. *Age Ageing*. 2002;31(4):247-254.
24. Sipila S et al. *Acta Physiol Scand*. 1996;156(4):457-464.
25. Sihvonen SE et al. *Gerontology*. 2004;50(2):87-95.
26. Sihvonen S et al. *Gerontology*. 2004;50(6):411-416.
27. Valtonen A et al. *Arch Phys Med Rehabil*. 2011;92(12):1944-1950.
28. Salpakoski A et al. *J Am Med Dir Assoc*. 2014;15(5):361-368.

29. Portegijs E et al. *Arch Phys Med Rehabil*. 2008;89(9):1667-1674.
30. Salpakoski A et al. *Biomed Res Int*. 2014;2014:289549.
31. Portegijs E et al. *J Aging Phys Act*. 2014;22(1):138-145.
32. Ngandu T et al. *Lancet*. 2015;385(9984):2255-2263.
33. Hillman CH et al. *Nat Rev Neurosci*. 2008;9(1):58-65.
34. Liu-Ambrose T et al. *Neurobiol Aging*. 2012;33(8):1690-1698.
35. Best JR et al. *Front Hum Neurosci*. 2014;8:353.
36. Voelcker-Rehage C et al. *Front Hum Neurosci*. 2011;5:26.
37. Rasinaho M et al. *Health Promot Int*. 2012;27(4):463-474.
38. Borella E et al. *Arch Clin Neuropsychol*. 2013;28(4):331-347.
39. Torgerson DJ. *BMJ*. 2001;322(7282):355-357.
40. Ho PM et al. *Circulation*. 2008;118(16):1675-1684.
41. Haskell WL et al. *Med Sci Sports Exerc*. 2007;39(8):1423-1434.
42. Leinonen R et al. *Scand J Med Sci Sports*. 2007;17(2):156-164.
43. Diggle P et al. 2nd Ed. Oxford: ed. Oxford University Press; 2002.
44. Zhu H et al. *Stat Med*. 2014;33(3):376-387.
45. Hortobagyi T et al. *Sports Med*. 2015.
46. Abellan van Kan G et al. *J Nutr Health Aging*. 2009;13(10):881-889.
47. Onder G et al. *J Gerontol A Biol Sci Med Sci*. 2005;60(1):74-79.
48. Menant JC et al. *PLoS One*. 2014;9(10):e109802.
49. Rantanen T et al. *BMC Public Health*. 2012;12:1018-2458-12-1018.
50. Paajanen T et al. *J Alzheimers Dis*. 2010;22(4):1089-1097.
51. Reitan RM. *Percept Mot Skills* 1958;8:271--276.
52. Koivisto K et al. *J Geriatr Psychiatry Neurol*. 1992;5(3):162-171.
53. Fan J et al. *J Cogn Neurosci*. 2002;14(3):340-347.
