# Supplementary material for: The effects of a physical and cognitive training intervention vs. physical training alone on older adults’ physical activity: A randomized controlled trial with extended follow-up during COVID-19
Source: PLoS One. 2021 Oct 13;16(10):e0258559. doi: 10.1371/journal.pone.0258559 (PMC8513828; doi:10.1371/journal.pone.0258559)
Supplement: S1 Table — (PDF) [file pone.0258559.s003.pdf]

S3 Table. The joint effects of executive functions on physical activity in pooled data.

	χ^2	p-value
EF_0		
PA_0	0.89	0.828
PA_6	9.46	0.024
PA_{12}	7.34	0.062
PA_{FU}	8.56	0.036
$PA_{COVID\text{-}19}$	7.49	0.058
$EF_{6\text{-}0}$		
PA_6	2.08	0.557
PA_{12}	2.87	0.412
PA_{FU}	4.28	0.233
$PA_{COVID\text{-}19}$	1.25	0.740
$EF_{12\text{-}0}$		
PA_{12}	1.41	0.704
PA_{FU}	1.74	0.629
$PA_{COVID\text{-}19}$	0.23	0.973

Note.

χ^2 = Wald chi-square test statistic

PA_0: Physical activity at baseline

PA_6: Physical activity at six months of the interventions

PA_{12}: Physical activity at twelve months of the interventions

PA_{FU}: Physical activity at one-year follow up

$PA_{COVID\text{-}19}$: Physical activity during COVID-19 restrictions

EF_0: Performance in the executive functioning tests at baseline

$EF_{6\text{-}0}$: Change in performance in the executive functioning tests from baseline to six months

$EF_{12\text{-}0}$: Change in performance in the executive functioning tests from baseline to twelve months
